# Experimental and Theoretical Investigation of the Reaction of C_2_H with Formaldehyde (CH_2_O) at Very Low Temperatures and Application to Astrochemical Models

**DOI:** 10.1021/acsearthspacechem.4c00188

**Published:** 2024-11-20

**Authors:** Kevin M. Douglas, Niclas A. West, Daniel I. Lucas, Marie Van de Sande, Mark A. Blitz, Dwayne E. Heard

**Affiliations:** † School of Chemistry, University of Leeds, Leeds LS2 9JT, U.K.; ‡ Leiden Observatory, Leiden University, P.O. Box 9513, Leiden 2300 RA, The Netherlands; § National Centre for Atmospheric Science (NCAS), University of Leeds, Leeds LS2 9JT, U.K.

**Keywords:** low-temperature kinetics, CRESU, astrochemistry, interstellar chemistry, rate theory calculations, MESMER

## Abstract

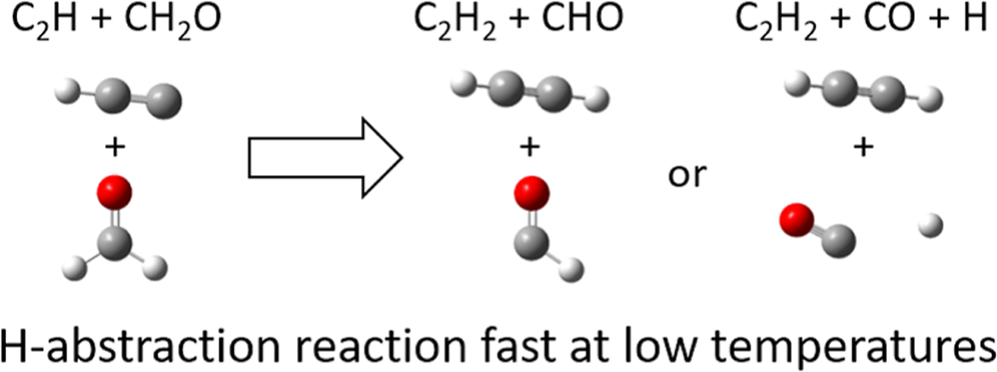

Rate coefficients for the reaction of C_2_H with CH_2_O were measured for the first time over the temperature range of 37–603 K, with the C_2_H radicals produced by pulsed laser photolysis and detected by CH radical chemiluminescence following their reaction with O_2_. The low temperature measurements (≤93 K) relevant to the interstellar medium were made within a Laval nozzle gas expansion, while higher temperature measurements (≥308 K) were made within a temperature controlled reaction cell. The rate coefficients display a negative temperature dependence below 300 K, reaching (1.3 ± 0.2) × 10^–10^ cm^3^ molecule^–1^ s^–1^ at 37 K, while only a slight positive temperature dependence is observed at higher temperatures above 300 K. Ab initio calculations of the potential energy surface (PES) were combined with rate theory calculations using the MESMER master-equation program in order to predict rate coefficients and branching ratios. The three lowest energy entrance channels on the PES all proceed via the initial formation of a weakly bound prereaction complex, bound by ∼5 kJ mol^–1^, followed by either a submerged barrier on the route to the H-abstraction products (C_2_H_2_ + CHO), or emerged barriers on the routes to the C- or O-addition species. MESMER calculations indicated that over the temperature range investigated (10–600 K) the two addition channels were uncompetitive, accounting for less 0.3% of the total product yield even at 600 K. The PES containing only the H-abstraction product channel was fit to the experimentally determined rate coefficients, with only a minor adjustment to the height of the submerged barrier (from −2.6 to −5.9 kJ mol^–1^) required. Using this new submerged barrier height, and including the subsequent dissociation of the CHO product into CO + H in the PES, rate coefficients and branching ratios were calculated over a wide range of temperatures and pressures and these used to recommend best-fit modified Arrhenius expressions for use in astrochemical modeling. Inclusion of the new rate coefficients and branching ratios in a UMIST chemical model of an outflow from an asymptotic giant branch (AGB) star yielded no significant changes in the abundances of the reactants or the products of the reaction, however, removal of the C-addition channel currently in the UMIST Rate22 database did result in a significant reduction in the abundance of propynal (HCCCHO).

## 1 Introduction

Much of the chemistry taking place around stars and planets occurs within the interstellar medium (ISM), a vast region of space between stars in a galaxy. Giant molecular clouds (planetary nebulae) in the ISM house most of the interstellar molecules, primarily provided by stellar winds from asymptotic giant branch (AGB) stars that inject molecules into star-forming regions of space.^1^ Over 300 molecules have now been identified in interstellar and circumstellar environments of space.^2^ Many of the molecules identified are termed complex organic molecules (COMs) since a large number of such molecules are carbon containing molecules of at least six atoms.^3^ Despite the development of complex arrays such as IRAM (Institut de Radioastronomie Millimétrique), NOEMA (Northern Extended Millimeter Array) and ALMA (Atacama Millimeter/submillimetre Array) enabling the interstellar observation of COMs with a variety of functional groups, the mechanisms for the formation and destruction of such molecules, and the relative importance of gas-phase versus gas-grain surface chemistry, remains largely unknown.^4−6^

Formation and destruction of interstellar molecules has been the subject of astrochemical research for many decades. Interstellar temperatures typically range from ∼10–100 K in gas clouds, although temperature up to ∼1000 K are possible due to the surge of supernova shock waves through these interstellar gas clouds. The three main categories of chemical reactions that contribute to molecule formation in the ISM are gas-phase ion-neutral and neutral–neutral reactions, and gas-grain surface chemistry.^1,7,8^ Ion-neutral reactions are reactions of charged species with a neutral molecule and have been well studied since many of these reactions are barrierless, exothermic reactions. Such reactions were perceived to dominate the chemistry of the ISM for many years as reactions between neutral molecules often have activation energy barriers and were therefore considered to be too slow at very low temperatures.^7,8^ However, there has been a growing number of gas-phase neutral–neutral reactions with small or no activation barriers that have been shown to be fast at low temperatures.^7,9^ Extrapolation of high temperature data for such reactions has in some cases been shown to be many orders of magnitude out,^10^ with the disagreement often due to a change in the chemical mechanism which governs the temperature dependence of the rate coefficient at low temperatures.^9,10^ Potential energy surfaces (PESs) that contain small and/or slightly submerged barriers to reaction may display a sharp increase in rate at low temperatures if the barriers are preceded by a weakly bound prereaction complex, often called a van der Walls complex. These weakly bound complexes in the entrance channel are formed with less energy under low temperature conditions, and as such their lifetime for dissociation back to reactants becomes sufficiently long that quantum mechanical tunnelling through barriers to products becomes competitive, resulting in sharp increases in rate coefficient with decreasing temperature.^10^ At higher temperatures dissociation of these complexes become rapid and they do not persist for long enough for tunnelling through to the barrier to occur; as such, the reaction can only proceed if the reactants possess enough energy to surmount the barrier, and the rate coefficient displays typical Arrhenius behavior at higher temperatures. Thus, these reactions display a U-shaped temperature dependence, with a minimum typically between 100 and 300 K. Examples of such behavior include the reactions of OH with oxygenated volatile organic compounds (OVOCs), such as OH with CH_3_OH.^11−14^ PESs with deeper submerged barriers or that are completely barrierless may also display a negative temperature dependence, consistent with a mechanism that involves the “capture” of the reactants by long-range intermolecular forces that become more prominent relevant to thermal energy as the temperature decreases.^7^ Examples of such behavior include the reaction of NH_2_ with NO,^15^ and the reaction of CH with O_2_.^16^ When using calculated PESs together with rate theory to predict temperature dependent rate coefficients, even minor changes to the height or width of small potential energy barriers can lead to large differences in the predicted rate coefficients. As such, it is important to consider the calculated PESs and rate coefficients alongside robust experimental data spanning a broad temperature range including low temperatures.

The current study focuses on the low temperature reaction of the ethynyl radical, C_2_H, with formaldehyde, CH_2_O. There have been no previous studies reporting experimental or theoretical rate coefficients for this reaction, however, the PES has been explored in a 2005 paper by Dong et al.^17^ who report a comprehensive PES showing a range of possible entrance channels, isomerization steps, and end products. Of the possible entrance channels, these fall into three groups; C_2_H abstracting an H atom from CH_2_O (H-abstraction), C_2_H adding to the C of CH_2_O (C-addition), and C_2_H adding to the O of CH_2_O (O-addition), and for each of these groups this may occur for either the terminal or central C on the C_2_H, resulting in 6 channels overall. As Dong et al.^17^ indicated that entrance channels involving the central C on the C_2_H were unfavorable due to the presence of significant barriers (>54 kJ mol^–1^), only the channels involving the terminal C on the C_2_H were considered in this study
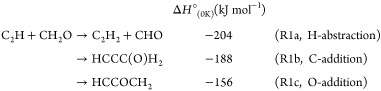
R1

Of these three channels, Dong et al.^17^ suggested that only the H-abstraction channel (R1a) would be important under interstellar conditions as it proceeds with only a submerged barrier, while the two addition channels contain emerged barriers. It is possible however that adsorption of the reactants onto interstellar dust or ice grains could result in the addition channels also becoming a barrier free process. Both the reactants (C_2_H and CH_2_O) and the likely products of reaction R1 (C_2_H_2_ and CHO) have been observed in several astrochemical environments, such as the ISM,^18−21^ dark clouds,^22−24^ and the stellar winds of AGB stars.^22,25,26^Reaction R1 may also be relevant to Titan^27,28^ and other planetary atmospheres,^29^ and to the combustion chemistry of organic molecules.^17^

In this paper we present experimentally determined rate coefficients for the reaction between C_2_H + CH_2_O, measured over the temperature range of 37–603 K. The low temperature kinetic measurements (<93 K) relevant to the interstellar medium (ISM) were made within a Laval nozzle expansion, while the higher temperature measurements were made within a temperature-controlled reaction cell. We also perform a theoretical investigation of the reaction, combining ab initio calculations with rate theory using the MESMER (master-equation solver for multienergy well reactions) program in order to calculate both rate coefficients and branching ratios over a range of temperatures and pressures. Finally, these new results have been input into a chemical network used for modeling the outflows of AGB stars.

## 2 Methodology

### 2.1 Experimental Study

Rate coefficient measurements for the reaction of C_2_H with CH_2_O were determined using a pulsed laser photolysis-chemiluminescence (PLP-CL) technique. The low temperature measurements between 37 and 93 K were carried out using a pulsed Laval nozzle apparatus, while a slow flow reaction cell was used for the higher temperature measurements between 308 and 603 K. The use of a Laval nozzle expansion for the study of low-temperature kinetics has been employed by this group to study a range of neutral–neutral reactions, including OH with unsaturated hydrocarbons^30^ and oxygenated volatile organic compounds,^12,14,31−33^ singlet methylene (^1^CH_2_) with a range of gases and hydrocarbons relevant to the atmosphere of Titan,^34,35^ CH,^36^ CN,^37^ and NH_2_^38^ with CH_2_O, and NH_2_ with CH_3_CHO^39^ and NO.^15^ As the Laval nozzle apparatus employed in this and previous studies has been discussed in detail elsewhere,^30,36^ only a brief description is given here.

Cylinders of ∼4% CH_2_O reagent were prepared by heating paraformaldehyde (Sigma-Aldrich, 95%) to generate CH_2_O, passing the nascent CH_2_O through a −10 °C trap to remove any impurities, and then adding the purified CH_2_O to an evacuated cylinder which was made up to ∼5 bar with bath gas (either He or N_2_). A more detailed procedure for CH_2_O generation can be found in our previous work.^40^ During experiments, gas from the CH_2_O/bath gas cylinder was then mixed with more bath gas, O_2_, and acetylene in a mixing manifold using calibrated Mass Flow Controllers (MFC; MKS Instruments) with a total flow rate of ∼2–5 slm (depending on the nozzle and bath gas used). Final mixtures of gases during experiments were ∼0.1–1% CH_2_O, <0.2% O_2_, <0.03% HCCH, and ∼99% bath gas. After collecting kinetics data with each gas mixture ratio, the remaining mixed gas in the mixing manifold was sampled into a UV absorption cell in order to measure the [CH_2_O] achieved, due to the tendency of CH_2_O to slowly repolymerize on the walls of the instruments and cause the MFC calibration to drift during a day. Our method of [CH_2_O] determination was described in detail previously^40^ and example UV absorption spectra are given in Figure S1 in the Supporting Information. After the mixing manifold, gas mixtures were pulsed at 10 Hz repetition frequency through 2 solenoid valves (Parker, series 9) into a 1 cm^3^ pre-expansion reservoir and then sent through a controlled expansion through one of several Laval nozzles into the vacuum chamber. The range of temperatures achieved by exchanging Laval nozzles and bath gas types was 37–93 K. The pressure in the vacuum chamber was monitored with a capacitance manometer (Leybold, type CTR90, 0–10 Torr), and controlled by adjusting the pumping speed of the dry screw pump (Edwards, GXS160/1750) used to evacuate the chamber.

The reaction of C_2_H + CH_2_O (R1) was initiated by the photolysis of acetylene with the ∼10 mJ cm^–2^ 193 nm ArF output of an excimer laser (Lambda Physik, LPX200) which was aligned colinearly with the Laval flow in counterpropagating directions.
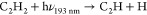
R2

The progression of the reaction was monitored via the rate of decay of CL generated via the reaction of C_2_H with O_2_.
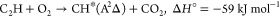
R3
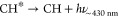
R4

**Figure 1:**
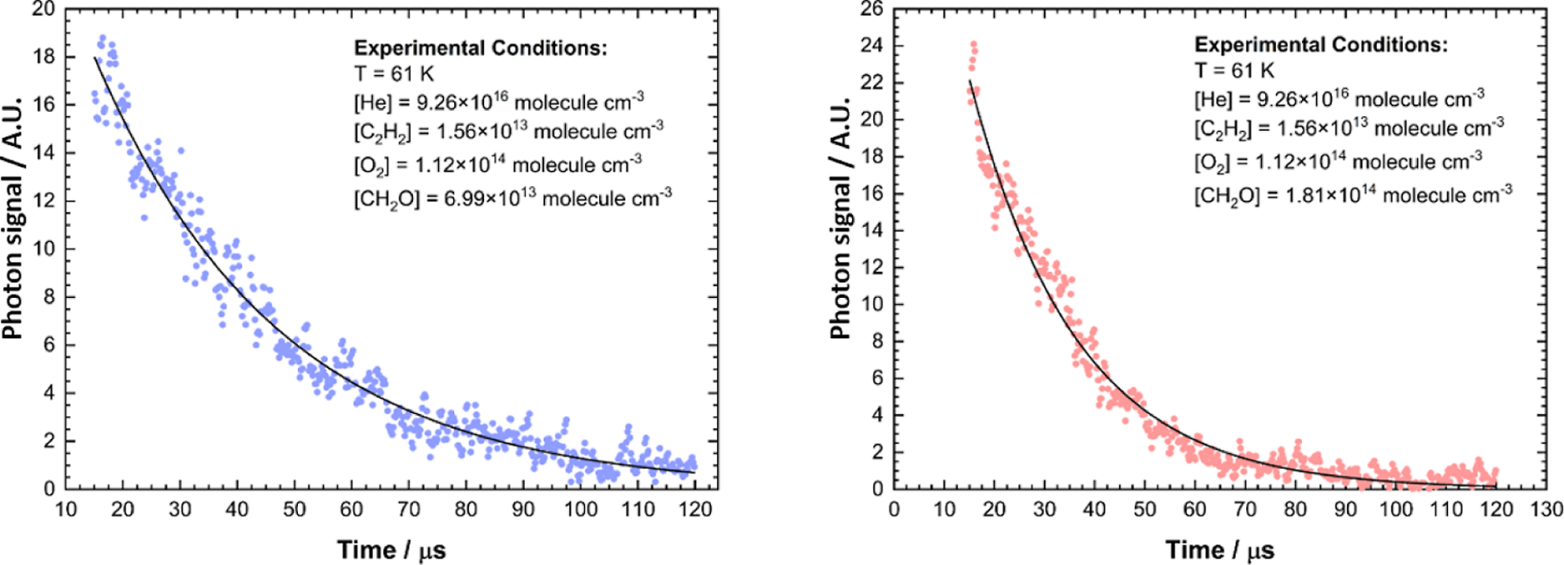
Typical CH(A^2^Δ) CL traces averaged over 1000 photolysis laser pulses upon photolysis of C_2_H_2_ in the presence of O_2_ and CH_2_O. Data were collected at 61 ± 6 K in He, [He] = 9.26 × 10^16^ molecules cm^–3^, [C_2_H_2_] = 1.56 × 10^13^ molecules cm^–3^; [O_2_] = 1.12 × 10^14^ molecules cm^–3^; left: [CH_2_O] = 6.99 × 10^13^ molecules cm^–3^; right: [CH_2_O] = 1.81 × 10^14^ molecules cm^–3^. Black lines are single exponential decays given by eq 1 which were fitted to the data.

The CL signal was collected via a series of lenses though an optical filter (Semrock, Brightline interference filter, λ_max_ = 427 nm, fwhm = 10 nm) and observed using a channel photomultiplier (CPM; PerkinElmer C1952P). The CPM was gated with a custom-built high-voltage gating box to remove laser scatter and the first ∼5 μs of signal. The CPM signal was digitized by an oscilloscope (PicoScope 6000) operated in 12-bit mode. Using 12-bit mode rather than the standard 8-bit mode was found not to affect the measured rates of CL decay. The decays were then averaged and saved onto a computer with the PicoScope 6 software. The timing of the Laval apparatus was maintained by a digital delay generator (Quantum Composers, 9520). A typical decay trace (Figure 1) was collected by averaging the observed CL from over 1000 photolysis laser pulses. The initial rise in the CL signal was due to the relatively fast photophysical relaxation processes of CH*, and was not quantified in this work.

One issue that may arise from this indirect method of monitoring the loss of C_2_H in our experiments is that photolysis of C_2_H_2_ at 193 nm may produce both ground and internally excited C_2_H radials (be that either electronic or vibrational excitation). This excited C_2_H would also react with O_2_ to form CH and produce chemiluminescence. As we are unable to distinguish between chemiluminescence signal produced from ground or excited state C_2_H, it is possible that some of the chemiluminescence signal we observe is from excited state C_2_H, and thus that the decay of the chemiluminescence signal is also from the decay of excited state C_2_H as well as ground state C_2_H. However, a number of measurements performed in a previous study by Chastaing et al.^41^ suggest that the rate coefficients determined in both this and the previous study are essentially independent of any internal excitation in the C_2_H radical. In this previous study, Chastaing et al.^41^ employ a similar experimental setup and method to measure low-temperature rate coefficients for the reactions of C_2_H with various unsaturated hydrocarbons. In both the present and earlier studies, if the CH chemiluminescence signal were from both ground and excited state C_2_H, it might be expected that the decay of the signal would be biexponential in nature, when in both studies the data can be accurately fit by a single exponential. Furthermore, in both studies three different bath gases are used, He, N_2_, and Ar, all three of which have been shown to electronically and vibrationally quench excited C_2_H at different rates,^78^ and again in all three bath gases the CH chemiluminescence decays can be fit accurately with a single exponential. In the earlier study by Chastaing et al.^41^ they also carried out experiments using both Ar and CO as a bath gas at *T* = 295 K. CO here was chosen as it can undergo an association reaction with C_2_H, and as such is likely to be very effective in relaxing vibrationally excited C_2_H. In these experiments, the rate coefficients obtained in both the Ar and CO bath gases were in good agreement, suggesting little or no interference from vibrationally excited C_2_H. Chastaing et al.^41^ also performed a series of experiments at *T* = 25 K in which the O_2_ concentration was varied by a factor of 3; despite this change in the concentration of O_2_, the rate coefficients determined were in good agreement. These results suggest that the rate coefficients determined in the present and earlier studies are not affected by internal excitation of the C_2_H following its formation.

Rate coefficient measurements made at 308 and 603 K were performed in slow flow reaction cell apparatus. The apparatus has been used for the measurement of a range of gas-phase reactions, and details of its operation can be found in previous publications (see for example see refs (42–44)). Reagent gases were prepared and delivered in the same manner as in the Laval experiments, and the same method of production and detection of C_2_H used. The pressure in the reaction cell was monitored by a Baratron capacitance manometer (Leybold Ceravac 0–10 Torr) and controlled by a valve on the exit line to the pump. Temperatures were measured close to the observation region using a K-type thermocouple.

Some additional experiments were also carried out looking at the dimerization of formaldehyde in some of our low-temperature expansions using laser-induced-fluorescence to monitor formaldehyde. Formaldehyde was observed by probing the A ^1^A_2_ (4_0_^1^) ← X ^1^A_1_ (0_0_^0^) transition around 353 nm.^45,46^ The probe laser beam was generated by frequency doubling the output of a Nd:YAG pumped dye laser (a Quantel Q-smart 850 pumping a Sirah Cobra-Stretch), and crossed the low-temperature expansion at a right angle. The nonresonant fluorescence at λ > 390 nm was discriminated using a long-pass Perspex filter, and observed by the CPM.

#### 2.1.1 Materials

He (99.9999%, BOC), N_2_ (99.999%, BOC), Ar (99.999 %, BOC) oxygen (99.998%, BOC), acetylene (99.998%, BOC), paraformaldehyde (95%, Sigma-Aldrich).

### 2.2 Theoretical Calculations

Ab initio electronic structure calculations were carried out using the Gaussian 09 program.^47^ Geometric structures of the stationary points (reactants, products, and intermediates including pre- and postreaction complexes, transition states, and adducts) were optimized at both the MP2/aug-cc-pVTZ^48,49^ and M062X/6-311+G(3df,2p)^50,51^ levels of theory. Rotational constants, harmonic vibrational frequencies, and zero point energies (ZPEs) were obtained from both levels of theory. Transition states (TSs) were found to have only one imaginary frequency, while for the reactants, products, and other intermediates all vibrational frequency values were positive. ZPEs obtained from the harmonic frequencies were corrected with a scaling factor corresponding to the level of theory employed (0.953 and 0.952 for the MP2 and M062X calculations respectively).^52^ Intrinsic reaction coordinate (IRC) calculations were performed for all identified TSs to verify that they are indeed saddle points on the minimum energy pathways connecting their respective local minima on the potential energy surface (PES). In order to obtain more accurate electronic energies, high-performance single-point energy calculations at the CCSD(T)^53^ level were also carried out using both the MP2 and M062X structures. These single point energies were extrapolated to the complete basis set (CBS) limit using the aug-cc-pVXZ basis sets (*X* = 2, 3, 4)^48^ and a mixed Gaussian/exponential extrapolation scheme proposed by Peterson et al.^54^

From the generated PES, statistical rate theory calculations were performed using the MESMER master equation solver program^55^ in order to predict temperature and pressure dependent rate coefficients and branching ratios. MESMER also has an inbuilt fitting feature in which various input parameters (e.g., the energies of the stationary points) can be adjusted in order to best fit experimental data such as rate coefficients or branching ratios. Further details of the parameters used in the MESMER program, such as the energy transfer parameters, Δ*E*_down_, are given in the MESMER input file attached as part of the Supporting Information. However, as discussed below, as the rate coefficient for the reaction between C_2_H and CH_2_O is effectively pressure independent (over pressure ranges of interest), the magnitude of the Δ*E*_down_ parameters does not play a role in the rate coefficients and BRs calculated.

## 3 Results

### 3.1 Experimental Rate Coefficients

**Figure 2:**
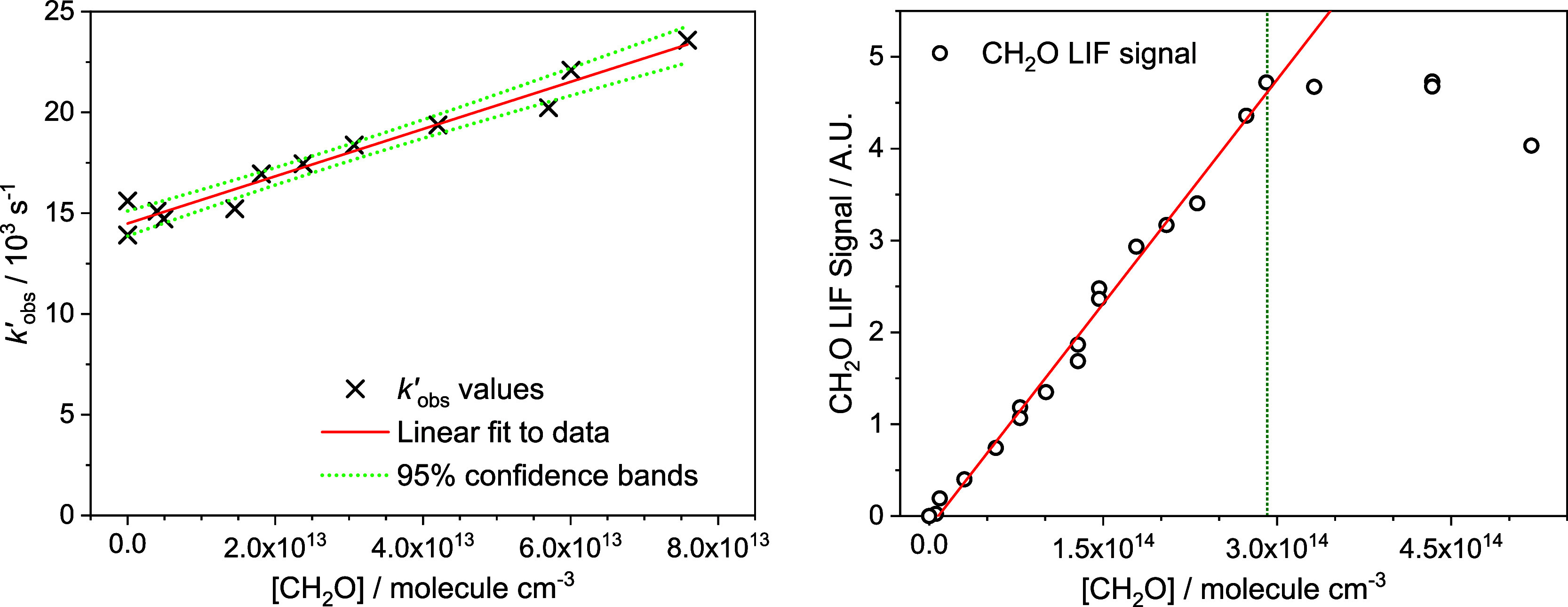
Left panel: Typical bimolecular plot of *k'*_obs_ vs [CH_2_O] for the reaction between C_2_H + CH_2_O (R1). The red line is a least-squares linear fit of eq 2 to the data, and the green dotted lines the upper and lower 95% confidence bands. Right panel: dimerization experiment observing CH_2_O LIF signal vs [CH_2_O]. The solid red line is a straight line fit to the data up to [CH_2_O] = ∼2.9 × 10^14^ molecules cm^–3^ (indicated with a green dotted line); the deviation from linearity above this point indicates significant dimer formation above this concentration. All data collected at *T* = 37 K and total He density of 5.1 × 10^16^ molecules cm^–3^.

Typical CH*(A^2^Δ) CL traces produced following the photolysis of C_2_H_2_ in the presence of O_2_ and CH_2_O can be seen in Figure 1. CL decays were subsampled such that 1 in every 63 data points in the temporal decay were utilized. The decay of the CL signal is the result of reaction of C_2_H with CH_2_O (R1), O_2_, and C_2_H_2_ present in the gas flow, as well as from diffusional loss. As experiments were carried out under pseudo-first-order conditions (i.e., [C_2_H] ≪ [CH_2_O], [O_2_], and [C_2_H_2_]), the loss of the C_2_H radical (and thus the CL signal) can be represented by a single exponential decay
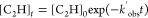
1and the pseudo-first-order rate coefficient, *k′*_obs_, is represented by eq 2
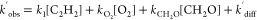
2where *k*_1_, , , and *k′*_diff_ are the rate coefficients for the reaction of C_2_H with C_2_H_2_, O_2_, CH_2_O, and diffusion of C_2_H out of the Laval expansion, respectively. As reported in previous publications, having C_2_H_2_ and O_2_ in slight excess over C_2_H allows for a steady-state approximation for the CH(A^2^Δ) concentration, where the CL intensity is directly proportional to the initial concentration of C_2_H.^41,56−58^ Photolytic generation of C_2_H followed by rapid relaxation caused a sharp rise in the CL signal at short times (≲2 μs) which was not resolved; hence only exponential loss that followed this initial increase in signal was used to retrieve the *k′*_obs_ values, with fitting of the traces beginning after ∼15 μs (where 0 μs represents the initiation time of the reaction with the pump laser), as shown in Figure 1. Plotting the *k′*_obs_ values obtained from the CL traces vs [CH_2_O] should then yield a straight line (eq 2) with a gradient equal to the bimolecular rate coefficient, *k*_1_, and an intercept the sum of the other pseudo-first order loss processes. An example of a typical bimolecular plot can be seen in Figure 2. As can be seen from Figure 2, there is a significant intercept in our bimolecular plot, which is the result of reaction of C_2_H with C_2_H_2_ and O_2_, and diffusional loss. In these experiments, the concentrations of C_2_H_2_ and O_2_ were minimized such that the majority of the intercept values can be explained by diffusion loss, with the rates typical of the low-temperature expansions employed. We have considered the possibility of interference from secondary chemistry resulting from the photolysis of CH_2_O in our experiments. There does not seem to be any cross-section measurements of CH_2_O at 193 nm in the literature, with the Mainz UV–vis Spectral Atlas having a window of no measurements between 180 and 200 nm, suggesting that the cross-section in this region is very small. Assuming a modest cross-section for CH_2_O of 5 × 10^–18^ cm^2^ molecule^–1^ at 193 nm would mean that less than 0.05% of CH_2_O is dissociated in our experiments per cm^3^, and this would require a rate coefficient from secondary radical chemistry to be on the order of 1 × 10^–7^ cm^3^ molecule^–1^ s^–1^ (orders of magnitude faster than gas kinetic) to be able to compete with the removal we observe.

It is possible that under the low-temperature conditions employed in this study, formaldehyde dimers (or even higher order oligomers) may form in our low-temperature expansions, resulting in a reduction in the amount of free formaldehyde monomer available. The presence of such dimers is typically observed as a curvature in the bimolecular plot of *k′*_obs_ versus [CH_2_O], as the dimers are unlikely to react at exactly twice the rate of the monomer (if the dimer reacts slower than twice as fast as the monomer, the bimolecular plot would curve downward, while if the dimer reacts faster than twice as fast as the monomer, the bimolecular plot would curve upward). However, in some cases it is not always obvious if there is a slight curvature in a bimolecular plot that may result in an over or underestimation of the determined rate coefficient. Therefore, rate coefficients were only collected in regimes in which no significant formaldehyde dimers were present, with the maximum [CH_2_O] to be used in each low-temperature expansion determined by one of two methods. In the first, the [CH_2_O] at which bimolecular plots for CH + CH_2_O curved over under equivalent pressure and temperature conditions^36^ was taken as the maximum [CH_2_O] to be used. In cases where CH + CH_2_O bimolecular plots were not available, separate formaldehyde dimerization experiments were carried out. In these experiments, the CH_2_O LIF signal was monitored as a function of [CH_2_O]. In regions in which no significant CH_2_O dimers were forming, the amount of CH_2_O monomer, and hence the LIF signal, would increase linearly with added CH_2_O. However, in regions in which CH_2_O dimers were forming, the amount of CH_2_O monomer present would be less than the [CH_2_O] added, and as such the CH_2_O LIF signal will begin to curve over with increasing [CH_2_O]. An example of such a CH_2_O dimerization plot can be seen in Figure 2, together with a bimolecular plot collected under the same conditions (*T* = 37 K, [He] = 5.1 × 10^16^ molecules cm^–3^) (these plots are overlaid in Figure S2 in the Supporting Information). As can be seen from Figure 2, the CH_2_O LIF signal begins to curve over at around [CH_2_O] = 2.9 × 10^14^ molecules cm^–3^, indicating that significant dimers are formed above this concentration; as such the maximum [CH_2_O] used in the kinetic experiment under the same conditions was kept well below this value.

**Figure 3:**
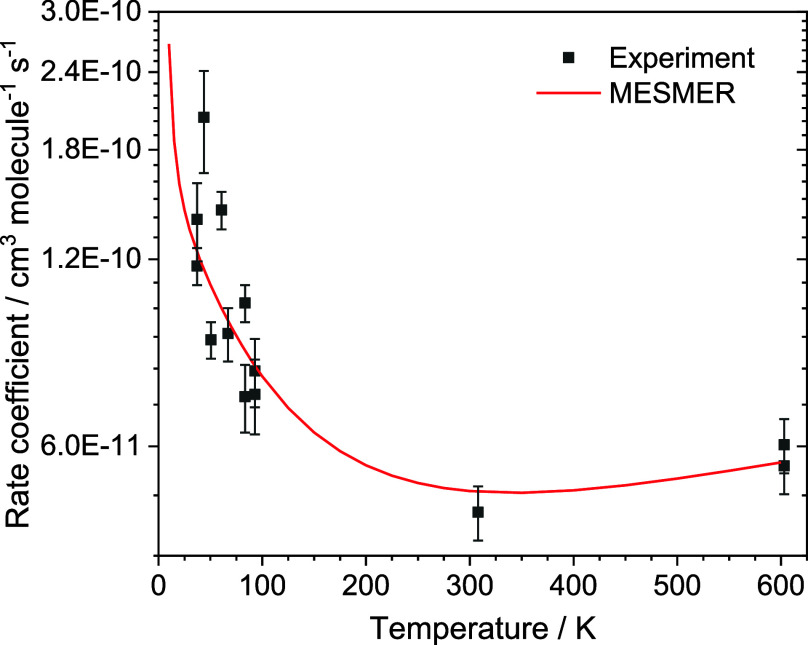
Temperature-dependent rate coefficients for the reaction of C_2_H + CH_2_O (R1), together with MESMER predicted rate coefficients following fitting of the PES to the experimental data points.

The bimolecular rate coefficients for the reaction of C_2_H with CH_2_O (R1) determined in this study are present in Table 1, and Figure 3. To the knowledge of the authors, these are the first measurements made for this reaction. There is some scatter in the experimentally determined rate coefficients, however it can still be clearly seen from Figure 3 that there is a slight negative temperature dependence in the low temperature rate coefficients for C_2_H + CH_2_O (at 300 K and below), consistent with the reaction pathway containing a slightly submerged barrier to reaction (−4.4 kJ mol^–1^ at the MP2 level of theory, see discussion below).

**Table 1:** Rate Coefficients, *k*(*T*), for the Reaction of C_2_H + CH_2_O Together with the Associated Experimental Conditions

*T*^a^/K	bath gas	*N*_total_^a^/10^16^ molecules cm^–3^	[O_2_]/10^14^ molecules cm^–3^	[C_2_H_2_]/10^13^ molecules cm^–3^	*k*_1_(*T*)^b^/10^–10^ cm^3^ molecule^–1^ s^–1^
37 ± 3	He	5.1 ± 0.7	0.52	0.71	1.2 ± 0.1
37 ± 3	He	5.1 ± 0.7	0.52	0.71	1.4 ± 0.2
44 ± 4	He	9.3 ± 1.0	1.0	1.4	2.0 ± 0.4
51 ± 4	He	7.1 0.8	0.68	1.0	0.89 ± 0.06
61 ± 6	He	9.3 ± 1.4	1.2	1.6	1.4 ± 0.1
67 ± 2	N_2_	2.9 ± 0.2	0.33	0.43	0.91 ± 0.09
83 ± 3	N_2_	7.4 ± 0.7	0.82	1.1	0.72 ± 0.09
83 ± 3	N_2_	7.4 ± 0.7	0.82	1.1	1.0 ± 0.07
93 ± 7	N_2_	5.3 ± 1.0	1.0	1.8	0.73 ± 0.10
93 ± 7	N_2_	5.3 ± 1.0	0.88	1.5	0.79 ± 0.10
308 ± 5	Ar	34 ± 2	3.4	23	0.47 ± 0.05
603 ± 5	Ar	82 ± 4	13	100	0.60 ± 0.06
603 ± 5	Ar	82 ± 4	25	140	0.56 ± 0.06

a Pitot tube impact pressure measurements along the axis of the nozzle were utilized to calculate average *T* and *N*_total_ values with thermodynamic relationships. Error bars were derived from the standard deviation of measurements along the axis of the nozzle.

b Uncertainties for each values of *k*_1_(*T*) reported at the 1σ level for the linear least-squares fitting of the pseudo-first-order rate coefficients *k′*_obs_ as a function of [CH_2_O].

## 4 Discussion

### 4.1 Theoretical Calculations

There has been one previous theoretical investigation of the reaction between C_2_H and CH_2_O by Dong et al.^17^ who produced a comprehensive potential energy surface showing a range of possible entrance channels, isomerization steps, and end products, mapped out at the B3LYP/6-311G(d,p) level of theory, as well as carrying out some higher level optimizations of some of the key species. Of the possible entrance channels, these fall into three groups; C_2_H abstracting an H atom from CH_2_O (H-abstraction), C_2_H adding to the C of CH_2_O (C-addition), and C_2_H adding to the O of CH_2_O (O-addition), and for each of these groups this may occur for either the terminal or central C on the C_2_H, resulting in 6 channels overall. As the focus of this work is on understanding the kinetics of reaction R1 at the low temperatures of the ISM, we need not consider entrance channels that contain large barriers above the energy of the reactants. As such, the three entrance channels involving the central C atom on the C_2_H can be immediately ruled out as outcomes in the ISM, as the work by Dong et al.^17^ indicated that these three channels are the least favorable energetically, and as such are only likely to occur at very high temperatures. Indeed, even the lowest energy entrance channel involving the central C atom of the C_2_H has a transition state that is 55 kJ mol^–1^ above the reactants.^17^ As such, only the entrance channels involving the terminal C atom on the C_2_H were considered in this work.

**Figure 4:**
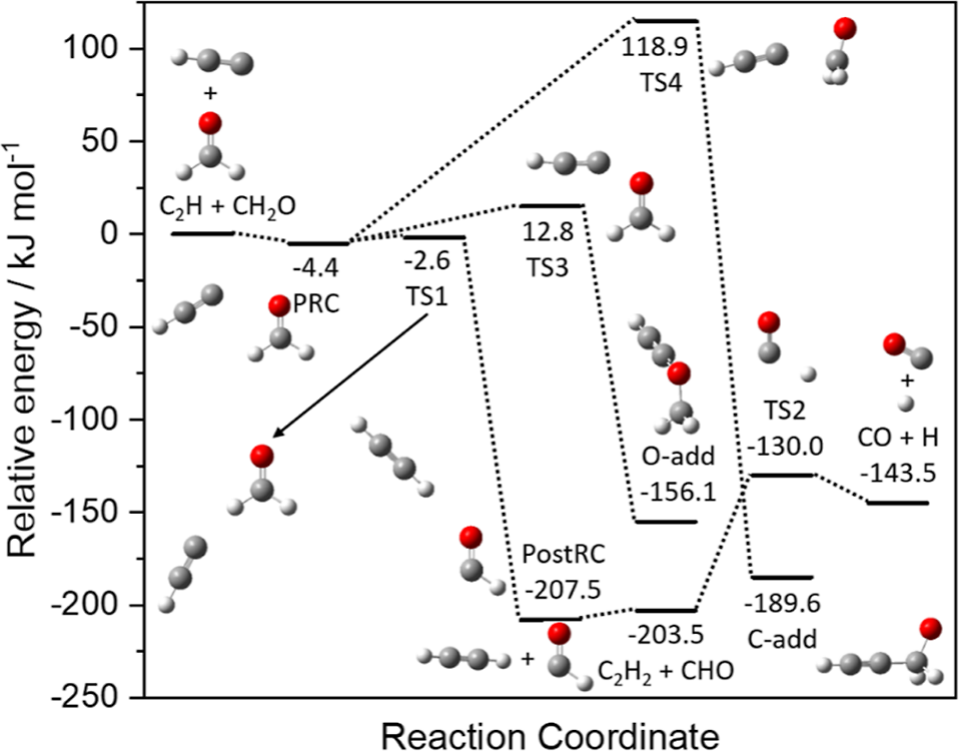
Potential energy surface for the reaction of C_2_H with CH_2_O showing the three entrance channels involving the terminal C atom on the C_2_H (R1a–R1c), together with the C_2_H_2_ + CHO (R1a1) and C_2_H_2_ + CO + H (R1a2) product channels, calculated at the CCSD(T)/aug-cc-pVXZ//MP2/aug-cc-pVTZ level of theory.

Figure 4 presents a potential energy surface for the reaction of C_2_H + CH_2_O, showing the three possible entrance channels involving the terminal C on the C_2_H; channel R1a is the H-abstraction channel forming either C_2_H_2_ + CHO (R1a1) or C_2_H_2_ + CO + H (R1a2), channel R1b is the C-addition channel forming HCCC(O)H_2_, and channel R1c is the O addition channel forming HCCOCH_2_. All three channels proceed via the initial association of a weakly bound prereaction complex (PRC).
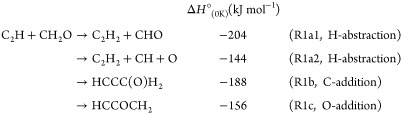


The PES displayed in Figure 4 was mapped out at both the MP2/aug-cc-pVTZ and M062X/6-311+G(3df,2p) levels of theory, with higher level single point energy calculations performed at the CCSD(T) level and extrapolated to the complete basis set limit on the optimized structures. Tables S1–S5 in the Supporting Information present the geometries of the stationary points together with the optimized Cartesian coordinates, vibrational frequencies, and rotational constants. Table S6 in the Supporting Information compares the relative energies of the stationary points calculated at the various levels of theory both in this study and by Dong et al. (2005),^17^ while those calculated at the CCSD(T) level are summarized in Table 2. In general, there is reasonable agreement between the energies calculated at different levels of theory, typically being within 20 kJ mol^–1^ of one another, with a few notable exceptions. For a number of stationary points, the MP2 energies calculated in this work are around 50 kJ mol^–1^ lower than many of the other calculations, however these discrepancies largely disappear when using the CCSD(T) energies calculated using the MP2 structures. Another notable discrepancy is in the B3LYP calculations by Dong et al.,^17^ which puts the depth of the PRC at around 10 kJ mol^–1^ deeper than the other levels of theory, and which could not locate transition state TS1, likely because the B3LYP method struggles to predict weak H-abstraction TSs as it usually underestimates long-range interactions. The CCSD(T) energy of the PRC calculated using the B3LYP structure is, however, again in good agreement with the other levels of theory. The largest discrepancy observed is in the calculated energy of the C-addition transition state, TS4, which ranges from 3 to 119 kJ mol^–1^ depending on the level of theory used, a range of 116 kJ mol^–1^. It should be noted that the lowest value (3 kJ mol^–1^) is from the B3LYP calculation, which as just discussed often underpredicts barrier heights. This discrepancy is primarily due to the CCSD(T) energies calculated using the MP2 and M062X structures in this work, which are around 90 kJ mol^–1^ higher than the MP2 and M062X energies. Surprisingly, this large increase in calculated energy when moving from a lower level of theory to CCSD(T) is not observed in the work of Dong et al.,^17^ despite there being little difference in the structures of TS4 calculated in the two studies. Although we are unable to explain why this large discrepancy exists, it is clear from both this study and the previous work of Dong et al.^17^ that TS4 presents as an emerged barrier on the PES with respect to the reactants. As such, channel R1b is likely to be uncompetitive no matter the absolute magnitude of TS4 when compared to channel R1a which has a submerged barrier. Indeed, preliminary MESMER calculations carried out with barrier heights for TS4 ranging from 12 to 119 kJ mol^–1^ (taken from the CCSD(T) energies for TS4 as calculated in this work and from Dong et al.^17^) indicated just this, with channel R1b only accounting for 0.2% of the total product yield at 600 K and only 0.02% at 300 K even when using the lowest barrier height (12 kJ mol^–1^). Channel R1c was also shown to be uncompetitive when compared to channel R1a, again due to presence of an emerged barrier TS3; even using the lowest CCSD(T) calculated energy for TS3 (13 kJ mol^–1^), preliminary MESMER calculations indicated that channel R1c still only accounted for <0.1% of the total product yield at 600 K, and <0.01% at 300 K.

**Table 2:** Comparison of the Relative Energies of the Stationary Points on the Potential Energy Surface for C_2_H + CH_2_O Shown in Figure 4 Calculated at Various Levels of Theory^g^

species	this work		Dong et al.^17^			ATcT^f^
	CCSD(T)//MP2^a^	CCSD(T)//M062X^b^	CCSD(T)//B3LYP^c^	CCSD(T)//MP2^d^	CCSD(T)//QCSID^e^	
PRC	–4.4	–5.4	–5.4	–3.35	–6.28	
TS1	–2.6	–2.0		–2.51	–2.93	
post RC	–207.5	–193.0	–188.7			
C_2_H_2_ + CHO	–203.5	–194.1	–186.2			–188.1
TS2	–130.0	–119.1	–129.7			
C_2_H_2_ + CO + H	–143.5	–133.8	–144.8			–127.3
TS3	12.8	31.24	36.8	22.18	36.40	
O-add	–156.1	–143.4	–124.3			
TS4	118.9	108.4	9.6	15.06	12.13	
C-add	–186.9	–176.4	–164.8			

a Levels of theory used in calculations: CCSD(T) energies extrapolated to the complete basis set limit using aug-cc-pVXZ (*X* = 2, 3, 4) and the MP2/aug-cc-pVTZ optimized structures.

b CCSD(T) energies extrapolated to the complete basis set limit using aug-cc-pVXZ (*X* = 2, 3, 4) and the M062X/6-311+G(3df,2p) optimized structures.

c CCSD(T)/6-311G(2d,p)//B3LYP/6-311G(d,p).

d CCSD(T)6-311+G(3df,2p)//MP2/6-311G(d,p).

e CCSD(T)6-311+G(3df,2p)//QCISD/6-311G(d,p).

f Reactions enthalpies (Δ*H*_f_° 0 K) taken from the Active Thermochemical Tables (ATcT).^62,63^

g Also included are literature values taken from the Active Thermochemical Tables (ATcT).^62,63^ All energies are given in kJ mol^–1^ and include zero-point energy.

As both channels R1b and R1c were shown to be uncompetitive at the temperatures explored in this study (10–600 K), the evolution of the C- and O-addition adducts via a series of isomerization steps and eventual dissociation into new product channels was not investigated in this study, and all further MESMER calculations were carried out using a reduced potential energy surface in which only the H-abstraction channel leading to products R1a1 and R1a2 were included. As discussed above, channel R1a proceeds via the initial formation of a weakly bound PRC, which is linked to a weakly bound postreaction complex (postRC) by a slightly submerged barrier (TS1). The energies of the PRC and TS1, as calculated in this study and by Dong et al.^17^ at the CCSD(T) level (see Table 2) are in very good agreement, ranging from −6.3 to −3.4 kJ mol^–1^ for the PRC, and from −2.9 to −2.0 for TS1. The postRC can then dissociate into the products C_2_H_2_ + CHO (channel R1a1), with any CHO formed with enough energy to overcome barrier TS2 also being able to dissociate into CO + H (channel R1a2). The structure of the PRC, in which the terminal C on the C_2_H points toward the electronegative O atom on the CH_2_O, may appear surprising. However, we were unable to locate any other stable PRCs at either the MP2 or M062X levels of theory. Dong et al.^17^ also only reported one stable PRC structure, that same as that reported in this study and found using the B3LYP level of theory. Other studies looking at C_2_H with other polar species such as H_2_O and NH_3_ also found stable PRC structures in which the terminal C on the C_2_H points toward the more electronegative O or N atoms.^59−61^ Ding et al.,^60^ using the B3LYP and MP2 levels of theory, and Carl et al.,^61^ using HF and B3LYP, both only found one PRC structure on the C_2_H + H_2_O surface, in which the terminal C on the C_2_H points toward the O atom on the H_2_O. Nguyen et al.,^59^ looking at the C_2_H + NH_3_ surface did find two PRCs; at both the B3LYP and CCSD(T) levels of theory the most stable PRC was found to be the one in which the terminal C on the C_2_H points toward the more electronegative N on the NH_3_, while in the more weakly bound complex the terminal C on the C_2_H pointed towards and interacted with all three of the terminal H atoms on the NH_3_. Another study by Bowman et al.^79^ looking at a range of C_2_H + X reactions (where X = NH_3_, H_2_O, HF, PH_3_, SH_2_, etc.) in which PESs were explored using the CCSD(T)-F12a method does appear to suggest that the PRC structures found have the terminal C on the C_2_H pointing toward the more positive terminal H atoms on the X species. However, this suggestion is only from a schematic in the graphical abstract, and as the optimized structures of the PRCs are not given we cannot comment further on this.

Comparing the PES to that of both the reactions of CN^37,64^ and OH^65−71^ with CH_2_O, similarities can be seen in the general profile and mechanisms. All three reactions possess an H-abstraction channel, which proceeds via the initial formation of a prereaction complex, followed by slightly submerged transition state to form products. For the OH system, earlier calculations placed this barrier as slightly emerged, however more recent calculations carried out using more robust methods tend to give lower values; the latest value is −5.7 kJ mol^–1^, as calculated by de Souza Machado et al.^67^ at the CCSD(T)/CBS level. For the CN system, two recent calculations^37,64^ put the barrier at only ∼1 kJ mol^–1^ submerged, however fitting of the CN + CH_2_O PES to experimental rate coefficients suggested that this barrier should in fact be slightly emerged, with the best fit to the data obtained with a barrier height of 4 kJ mol^–1^.^37^ The CN system also contains the C- and O-addition species comparable to those formed via channels R1b and R1c in the current work. Despite an earlier theoretical study by Tonolo et al.^64^ suggesting that the C-addition channel is barrierless, a more recent study by West et al.^37^ found a significant barrier of 50 kJ mol^–1^, making this channel the least competitive in the CN + CH_2_O system, while the O-addition barrier was found to be 33 kJ mol^–1^.

### 4.2 Rate Theory Calculations

In addition to predicting rate coefficients and branching ratios, the MESMER program can also obtain a best-fit to available experimental data by allowing various parameters used in the rate theory calculation or features of the PES itself to be adjusted. We have identified five key parameters that may have a significant effect on the calculated rate coefficients, and as such are suitable to being adjusted when fitting to the experimental rate coefficients. These are (i and ii) the inverse Laplace transform (ILT) parameters A and n for the initial association reaction of C_2_H with CH_2_O to form the PRC, which takes the form of a modified Arrhenius function *A*(*T*/300)^*n*^ (with the activation energy being set to zero for the barrierless process). (iii) The energy (or depth) of the PRC well. (iv) The energy (or height) of the barrier to H-abstraction (TS1). (v) The imaginary frequency of the H-abstraction barrier (TS1) which affects the rate of tunnelling through the barrier. For pressure dependent reactions, the energy transfer parameters for collisions with the bath gas (Δ*E*_down_) would also be expected to affect the calculated rate coefficients, however as discussed below we demonstrate that the reaction between C_2_H and CH_2_O is pressure independent. Fitting to experimental data was carried out using the PES surface calculated in this work at the CCSD(T)/aug-cc-pVXZ//MP2/aug-cc-pVTZ level of theory, although as previously mentioned there is very good agreement between the energies of the key features on this surface (the depth of the PRC and the hight of TS1) and the other surfaces calculated at the CCSD(T) level both in this work and by Dong et al. (see Table 2). MESMER struggles with predicting or fitting to rate coefficients at low temperatures when the PES contains deep wells, as it must calculate both the forward and reverse reactions into and out of the wells. When a well is deep and the temperature low, the rate out the well becomes very slow, eventually reaching the precision limit of the computation. As such, fitting of the PES was done using a reduced PES containing only channel R1a1 (i.e., with only the reactants, the PRC, TS1, and the postRC; see Figure S3b in the Supporting Information). Using this reduced surface, the postRC could be set to a sink (irreversible one way process to form it), removing the deep well problem and enabling MESMER to both predict and fit to rate coefficients even down to 10 K. Once the fitting had been completed, channel R1a2 was reintroduced to the PES (see Figure S3c in the Supporting Information) so that the branching ratio between the two channels could be determined at higher temperatures (≥80 K), and the rate coefficients calculated both with and without channel R1a2 were shown to be within 1% of each other. The experimental data used in the fitting are the rate coefficients and errors presented in Table 1. Initially each parameter was fitted independently (except the A and n parameters which were always floated together) in order to assess its effect on the predicted rate coefficients, before allowing several parameters to float at once. Both the depth of the PRC well and the imaginary frequency were shown to have little effect on the predicted rate coefficients, with both being poorly defined when fitting to the experimental data. As TS1 lies below the energy of the reactants and is only marginally higher in energy than the PRC, H atom tunnelling does not play a large part in the determined rate coefficients, and as such neither does the imaginary frequency of TS1. Thus, the imaginary frequency was fixed at its calculated value in the final fitting. For the PRC, as the reaction must proceed over TS1 to form the products, increasing the depth of the PRC that lies immediately before TS1 has little effect on the rate coefficients, make it poorly defined when fitting to the experimental data. Only the A and n ILT parameters and the energy of TS1 were shown to significantly affect the calculated rate coefficients (although as discussed later when only the A and n parameters are floated they are not well-defined by the fitting), and as such a fit allowing both to float was carried out. Rather than fixing the energy of the PRC in this final fitting, which could result in it being higher in energy than TS1, the absolute energy difference between the PRC and TS1 was fixed at the calculated value, meaning that as the energy of TS1 could move up or down during the fitting, the energy of the PRC would always remain 1.8 kJ mol^–1^ lower in energy.

The values of the parameters A and n and the energy of TS1 returned from fitting to the experimental data is given in Table 3, while Figure 2 and Table S7 compares the calculated rate coefficients with the experimental data. As can be seen from Table 3, only a minor adjustment to the height of TS1 was required when fitting to the data, with the ZPE of the submerged barrier moving down from −2.6 to −5.9 kJ mol^–1^, a decrease in height of 3.3 kJ mol^–1^ and well within the error of ∼5 kJ mol^–1^ expected of CCSD(T) calculations.^72−74^ Both the ZPE of TS1 and the *A* factor of the ILT were well-defined by the fitting, with relative errors of <25%, while the return of a small negative value for n indicates that the initial association of the C_2_H with CH_2_O has only a slight negative temperature dependence. Looking at Figure 3 and Table S7, it can be seen that these returned parameters give a satisfactory fit to the experimental rate coefficients over the entire temperature range (37–603 K), with most of the calculated values being within 20% of the experimental, and the average % difference between the two values being 14%. To highlight the importance of the height of TS1 to the calculated rate coefficients, several additional fits were carried out using PESs with energies from four different CCSD(T) calculations (form both this work and Dong et al.^17^) and in which only the ITL parameters were allowed to float. Table 3 gives the returned A and n parameters from these fits, together with the goodness of each fit (the χ^2^ values). As can be seen from Table 2, the quality of the fits when TS1 is fixed are considerably worse than when it is allowed to float, and the returned A and n parameters are ill defined with substantial errors. Similar A parameters were returned from the fits to each surface, which is unsurprising considering the similarity in the energies of the PRC and TS1 between the four surfaces, however the returned n parameters are scattered, with one fit suggesting a moderate positive temperature dependence, one a moderate negative temperature dependence, and two suggesting little temperature dependence, although due to the large returned errors all four agree within mutual error limits.

**Table 3:** Parameters Adjusted during Fitting to the Experimental Data in the Rate Theory Calculations Together with the Values Obtained from the Fitting (Errors are 1σ) when Using PESs Calculated at Various Levels of Theory^f^

parameter	fitting of PES^b^ to experimental data	this work		Dong et al.	
		CCSD(T)//MP2^b^	CCSD(T)//M062X^c^	CCSD(T)//MP2^d^	CCSD(T)//QCISD^e^
*A*/10^–10^ cm^3^ molecule^–1^ s^–1^	1.20 ± 0.30	7.79 ± 5.11	7.56 ± 9.78	7.61 ± 5.06	7.51 ± 4.71
*n*	–0.040 ± 0.058	0.054 ± 0.230	–0.293 ± 0.403	0.053 ± 0.231	0.238 ± 0.235
TS1 ZPE/kJ mol^–1^	–5.90 ± 0.81	–2.6 (fixed)	–2.0 (fixed)	–2.5 (fixed)	–2.9 (fixed)
χ^2^ (degrees of freedom)^a^	4.6 (10)	19.4 (11)	33.9 (11)	22.6 (11)	13.9 (11)

a Values given are χ^2^/degrees of freedom (number of experimental points minus number of parameters, given in brackets).

b PES calculated at the CCSD(T)/aug-cc-pVXZ//MP2/aug-cc-pVTZ level of theory.

c PES calculated at the CCSD(T)/aug-cc-pVXZ//M062X/6-311+G(3df,2p) level of theory.

d PES calculated at the CCSD(T)6-311+G(3df,2p)//MP2/6-311G(d,p) level of theory.

e CCSD(T)6-311+G(3df,2p)//QCISD/6-311G(d,p) level of theory.

f See text for details.

Using the A and n parameters and the energy of TS1 obtained from fitting to the experimental data, we have calculated rate coefficients over the temperature range of 10–600 K. As discussed above, due to the deep well problem, these rate coefficients were calculated using a reduced PES containing only channel R1a1 (see Figure S3b), but still represent the total removal kinetics of C_2_H with CH_2_O. The temperature dependence of the calculated rate coefficients can be seen in Figure 3 and Table S7. These rate coefficients predicted by MESMER are in effect the zero pressure rate coefficients applicable to the interstellar medium (ISM), confirmed by the rate coefficients calculated between 10 and 600 K being pressure independent over the range 1 × 10^11^ to 1 × 10^21^ molecules cm^–3^. Due to the unique shape of the *k* vs *T* plot, the rate coefficient data were parametrized at both high (≥300 K) and low (≤300 K) temperatures, giving
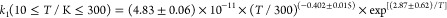
3
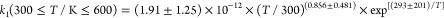
4

**Figure 5:**
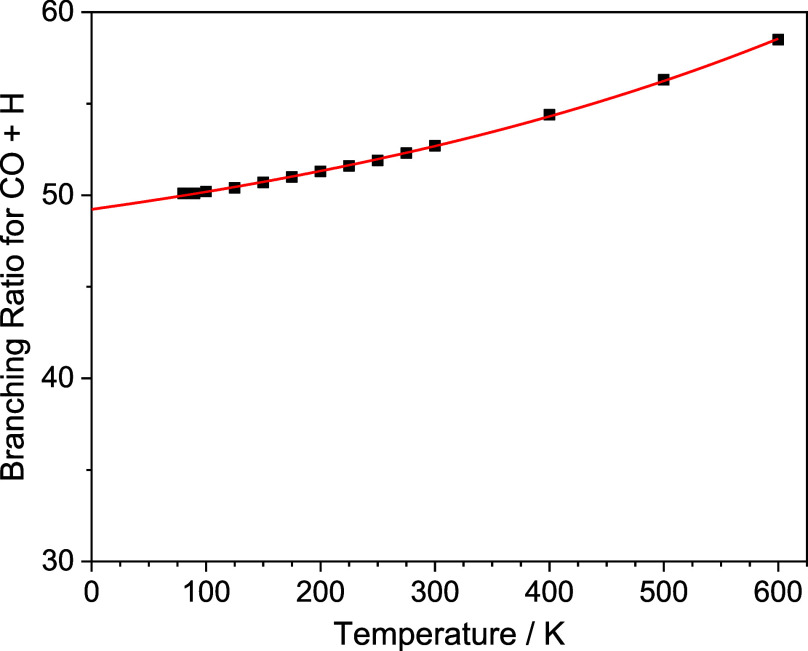
% branching ratios for channel R1a2 (C_2_H_2_ + CO + H) calculated down to 80 K (black squares), together with a single exponential fit to the data (solid red line) to allow extrapolation down to 0 K.

The branching ratio (BR) between channels R1a1 and R1a2 has also been calculated using MESMER, over the temperature range 80–600 K. Both channels R1a1 and R1a2 proceed via H-abstraction to produce C_2_H_2_ + CHO (R1a1), however in channel R1a2, the CHO product promptly dissociates to form CO + H (R1a2). The assumption in this calculation is that the energy in the C_2_H_2_ and CHO products is distributed statistically. In the case of CHO, there is an effective amount of energy where above this leads to “instant” dissociation, which can be consider well-skipping as the H + CO products form at the same rate as the C_2_H loss. Due to the deep well problem discussed above, BRs could only be calculated down to 80 K, however, plotting the BR for channel R1a2 against temperature (see Figure 5), it can be seen that the BR appears to be approaching a limit as we move toward 0 K. We have fit a single exponential to the BR vs temperature plot in order to be able to extrapolate the BR down to 0 K (see solid red line Figure 5 and Table S8). As can be seen from Figure 5, channel R1a2 accounts for almost 60% of the product yield at 600 K, decreasing to just below 50% of the product yield at 0 K. The BR for channel R1a2 calculated by MESMER can be thought of as the instant CO + H product, which is formed on the same time scale as the C_2_H and CH_2_O reactants are lost, and is largely dependent on how the reaction enthalpy is distributed between the C_2_H_2_ and CHO products, although the small positive temperature dependence does indicate a small thermal contribution to the BR.

**Figure 6:**
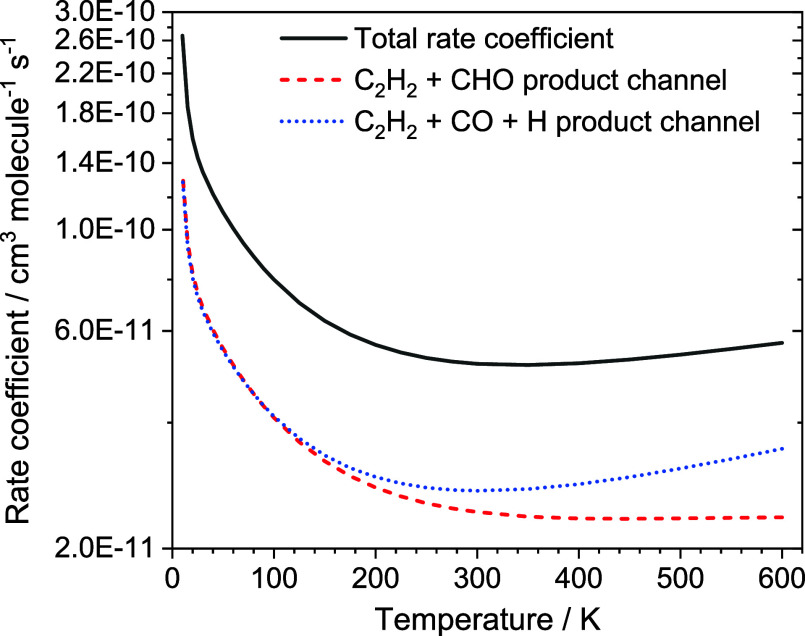
Temperature dependent rate coefficients for; solid black line: total C_2_H + CH_2_O removal rate (*k*_1_); red dashed line: product channel C_2_H_2_ + CHO (*k*_1a1_); blue dotted line: product channel C_2_H_2_ + CO + H (*k*_1a2_).

These BRs for the instant CHO or CO + H product have been shown to be pressure independent over the range of 1 × 10^11^ to 1 × 10^21^ molecules cm^–3^, and as such can be taken as the zero-pressure BRs relevant to the ISM. It should be noted however that at elevated temperatures and pressures, any instant CHO produced may also go onto dissociate into CO + H on longer time scales, and as such astrochemical models should include the dissociation reaction CHO → CO + H. By applying these calculated BRs to the calculated rate coefficients, we have produced channel specific rate coefficients which can be seen in Figure 6 and Table S8, and parametrized as
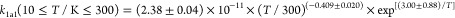
5
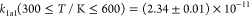
6
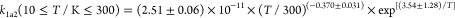
7
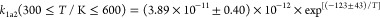
8

### 4.3 Astrochemical Implications

The rate coefficients and product channels for the C_2_H + CH_2_O reaction were added to a chemical network used for modeling the outflows of AGB stars. The gas phase chemistry in this network is based on the Rate22 release of the UMIST database for Astrochemistry.^75^ The Rate22 network does include the reaction between C_2_H + CH_2_O, however the products given are the C-addition (followed by H-elimination) products HCCCHO + H, and rather than there being any temperature dependence to the rate coefficient, it is set to 1 × 10^–10^ cm^3^ molecule^–1^ s^–1^ over the temperature range 10–300 K. The results of this study indicate that this channel is unviable at the low temperatures of the ISM due a barrier on the PES to C-addition. The AGB outflow model is based on the publicly available UMIST CSE model.^75,76^ The outflow is assumed to be spherically symmetrical, with an expansion velocity of 15 km s^–1^ and a stellar mass loss rate of 10^–5^ solar masses (*M*_sun_) per year. The temperature of the outflows as a function of distance *r* is parametrized as
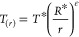
9where T* and R* are the stellar temperature and radium respectively, and ε is the power law exponent, set at ε = 0.7. Both C-rich and O-rich outflows were considered, with the initial abundances of parents species taken from Van de Sande and Millar.^77^

For each of the C-rich and O-rich outflows, model runs were carried out turning on and off both the HCCCHO + H product channel in the original Rate22 network and the new product channels (R1a1 and R1a2) as determined in this work, resulting in a total of 4 model outputs for each type of outflow. If the HCCCHO + H product channel was turned on, its rate coefficient was set to 1 × 10^–10^ cm^3^ molecule^–1^ s^–1^ as given in the Rate22 network, while if the product channels R1a1 and R1a2 were turned on their rate coefficients were set to those given in eqs 5–8 as determined in this study.

**Figure 7:**
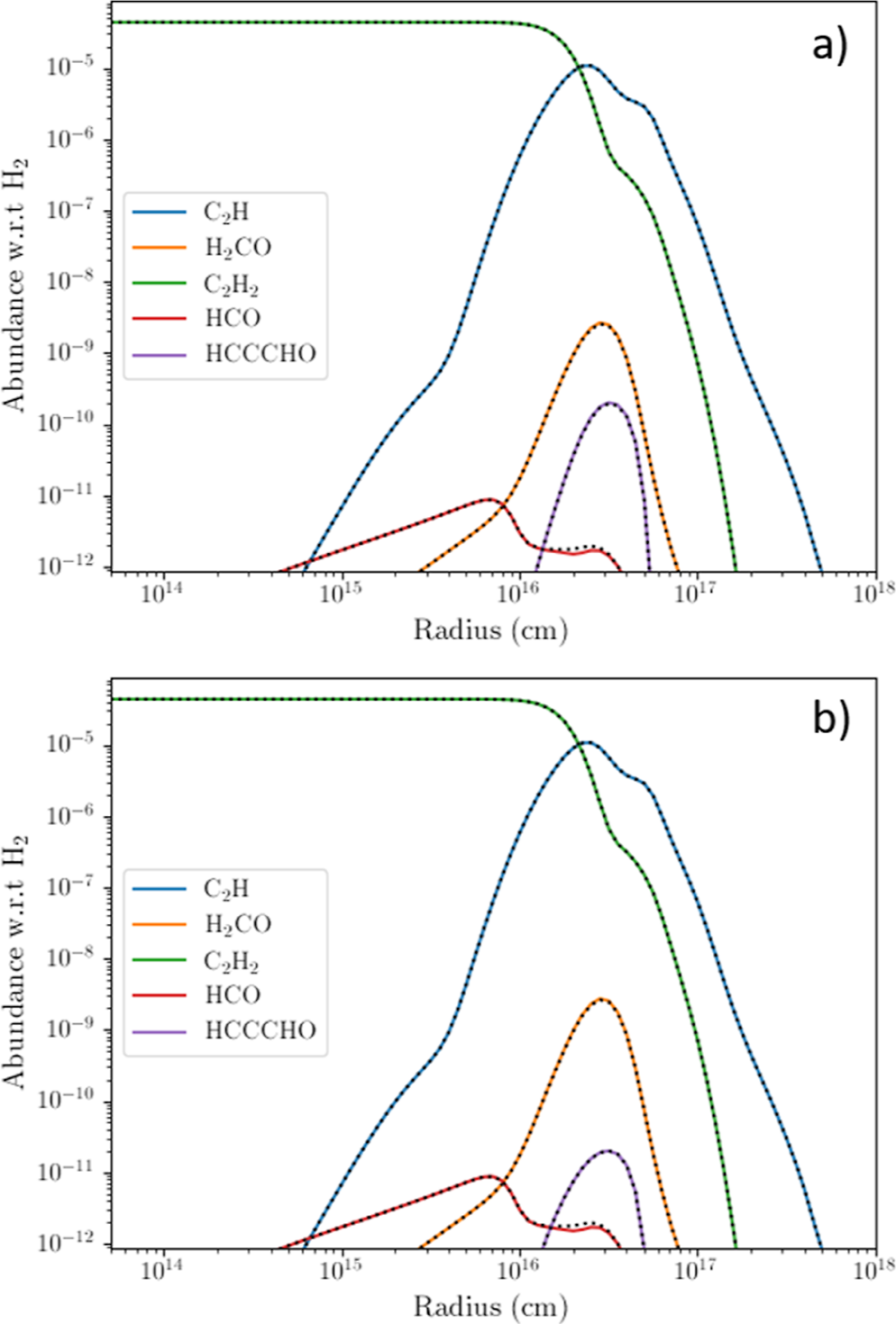
Fractional abundance profiles relative to H_2_ for C_2_H and CH_2_O (reactants), C_2_H_2_ and CHO (products of channel R1a1), and HCCCHO (product in original Rate22 network) throughout the C-rich AGB outflow. The HCCCHO + H product channel was turned on in panel (a) and off in panel (b). In both panels the coloured curves relate to the profiles in which the new product channels R1a1 and R1a2 were turned off, while the black dotted curves relate to profiles in which they were turned on. The overlap of the coloured and black dotted curves shows that our new rate coefficients do not change the observed abundances, except for that of HCO for which there is small difference, however this would be too small to be observable. The product species CO and H, for which no change was observed in their abundances regardless of model run, are excluded from the figures for clarity, as their abundances are significantly higher than the other species shown.

Figure 7 presents the model results for the C-rich outflow. We find that in both the C- and O-rich outflows, inclusion of the new rate coefficients and product branching ratios did not affect the abundances of the reactants (C_2_H and CH_2_O) or the products (C_2_H_2_, CHO, CO, and H) of reaction R1, except for a very marginal increase in the abundance of HCO in the C-rich outflow that would be too small to be observable. However, turning off the HCCCHO + H product channel that was included in the original Rate22 network did have an appreciable effect on the HCCCHO abundance, which fell by around an order of magnitude in both the C- and O-rich outflows.

## 5 Conclusions

Rate coefficients, *k*_1_(*T*) for the reaction between C_2_H and CH_2_O have been measured for the first time over the temperature range 37–603 K. The low temperatures relevant to many astrochemical environments were achieved using a pulsed Laval nozzle expansion, while the room temperature and above measurements were made using a reaction cell. C_2_H radicals were produced by the pulsed laser-photolysis of C_2_H_2_, and their removal in the presence of CH_2_O observed by CH radical chemiluminescence following their reaction with O_2_. The reaction exhibits a clear negative temperature dependence below 300 K, while above this temperature a small increase in rate coefficient is observed. Ab initio calculations of the potential energy surface (PES) were carried out, focusing on the three most thermodynamically favorable entrance channels, and these combined with rate theory calculations using the MESMER program. For all three entrance channels, the reaction proceeds via the initial formation of a weakly bound (∼5 kJ mol^–1^) prereaction complex (PRC). The two additional channels presented with emerged barriers, making them uncompetitive when compared to the H-abstraction channel on which there is only a submerged barrier; at 600 K the addition channels accounted for <0.3% of the total product yield, and at the low temperatures of the interstellar medium they are completely unviable. The PES containing only the H-abstraction channel was fit to the experimentally determined rate coefficients, requiring only a minor adjustment to the to the height of the submerged barrier (from −2.6 to −5.9 kJ mol^–1^). Using the submerged barrier height from the fitting, and including the subsequent dissociation of the CHO product into CO + H (channel R1a2) in the PES, rate coefficients and branching ratios were calculated over a wide range of temperature and pressures. At low temperatures the product yield for channel R1a2 is around 50%, and slowly increases to around 60% at 600 K. We have fitted modified Arrhenius expressions to the calculated rate coefficients and branching ratios, providing recommended best-fit expressions for use in astrochemical models. These parametrizations of *k*_1_ were used as an input to an astrochemical model of the outflow from an AGB star. No significant changes in the abundances of the reactants (C_2_H and CH_2_O) or products (C_2_H_2_, CHO, CO, and H) of R1 were observed following its inclusion in the model run. However, removal of the C-additional channel currently in the UMIST Rate22 database did result in a significant reduction in the abundance of HCCCHO.

## Supporting Information Available

Figures S1–S3: UV absorption spectrum of CH_2_O, a dimerization plot of CH_2_O, and the various PESs used in the MESMER calculations. Tables S1–S6: results of the electronic structure calculations (optimized structures, Cartesian coordinates, vibrational frequencies, and rotational constants, and relative energies). Tables S7 and S8: experimental and calculated rate coefficients and branching ratios (PDF)
Also attached separately is an example MESMER input file (PDF)
