# Supplementary material for: Experimental and Theoretical Investigation of the Reaction of C2H with Formaldehyde (CH2O) at Very Low Temperatures and Application to Astrochemical Models
Source: ACS Earth Space Chem. 2024 Nov 20;8(12):2428–41. doi: 10.1021/acsearthspacechem.4c00188 (PMC11664655; doi:10.1021/acsearthspacechem.4c00188)
Supplement: Supplementary file 1 — sp4c00188_si_001.pdf [file sp4c00188_si_001.pdf]

Supplementary Information

An Experimental and Theoretical Investigation of the Reaction of C_2H with formaldehyde (CH_2O) at Very Low Temperatures and Application to Astrochemical Models

Kevin M. Douglas[a*], Niclas A. West[a], Daniel I. Lucas,[a‡], Marie Van de Sande[b], Mark A. Blitz[a,c], Dwayne E. Heard[a*]

[a]*School of Chemistry, University of Leeds, Leeds, LS2 9JT, UK*

[b]*Leiden Observatory, Leiden University, P.O. Box 9513, 2300 RA Leiden, The Netherlands*

[c]*National Centre for Atmospheric Science (NCAS), University of Leeds, Leeds, LS2 9JT, UK*

[*]corresponding authors. Email: k.m.douglas@leeds.ac.uk; d.e.heard@leeds.ac.uk

[‡]now at *School of Chemistry, University of Birmingham, Edgbaston, B15 2TT, UK*

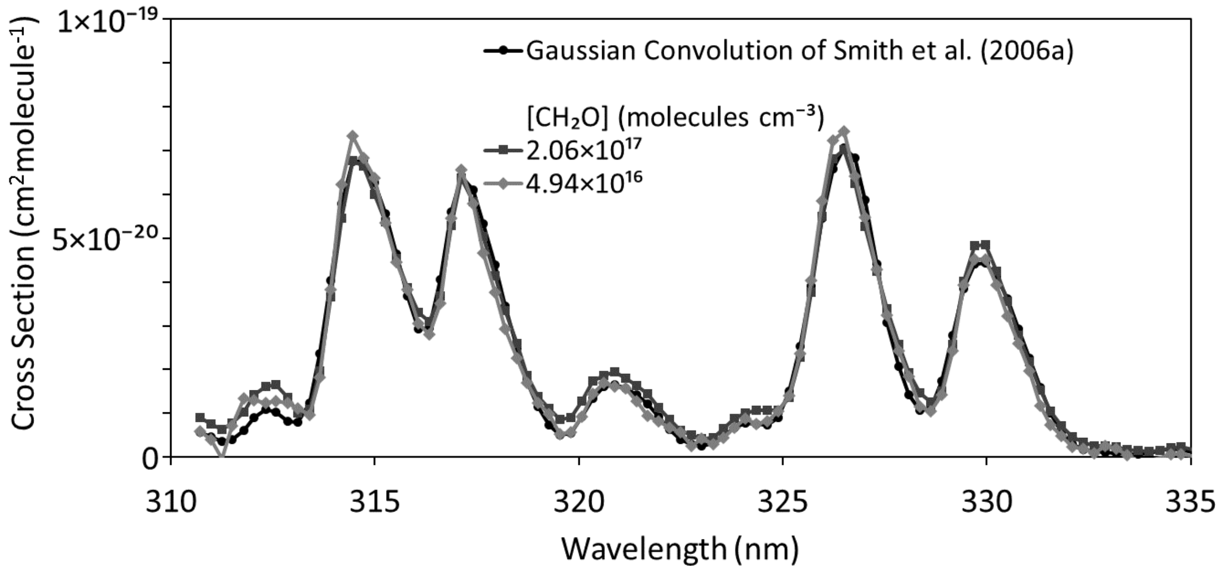

Figure S1. Convolution of a high resolution spectrum from Smith, *et al.*[1] with a gaussian of 0.75 nm FWHM along with experimentally measured CH_2O absorption cross sections which were utilized to verify the [CH_2O] utilized in C_2H + CH_2O experiments. A detailed description of the method utilized can be found in West, *et al.*[2]

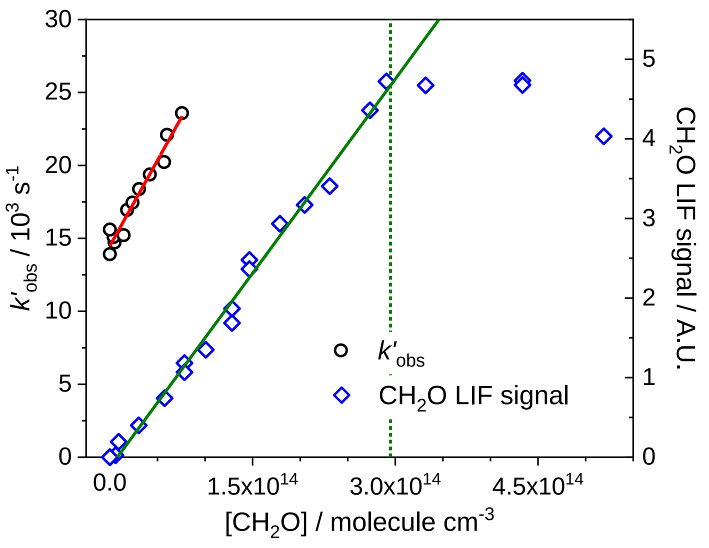

Figure S2. Left axis: bimolecular plot of k'_{obs} *vs*. [CH_2O] for the reaction between C_2H + CH_2O (R1). The red line is a straight line fit to the data (Eq. 2). Right axis: dimerization experiment observing CH_2O LIF signal *vs*. [CH_2O]. The solid green line is a straight line fit to the data up to [CH_2O] = ~2.9×10^{14} molecule cm^{-3} (indicated with a green dotted line); the deviation from linearity above this point indicates significant dimer formation above this concentration. All data collected at T = 37 K and total He density of 5.1×10^{16} molecule cm^{-3}.

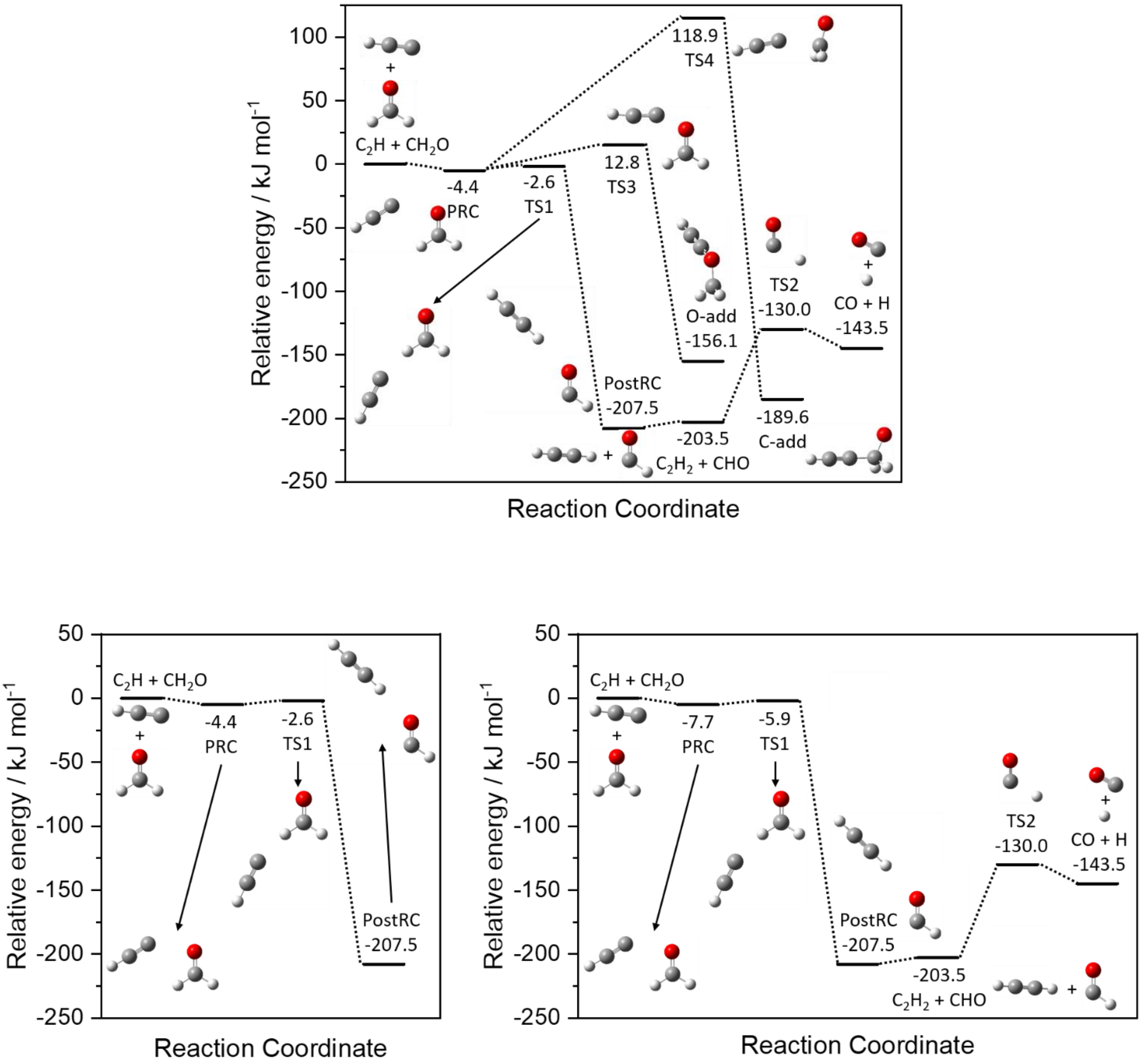

Figure S3. Various potential energy surfaces for the reaction of C_2H with CH_2O used in MESMER calculations, calculated at the CCSD(T)/aug-cc-pVXZ//MP2/aug-cc-pVTZ level of theory, showing; top panel: the three entrance channels involving the C-atom on the C_2H attaching the CH_2O (R1a – R1c), together with the C_2H_2 + CHO (R1a1) and C_2H_2 + CO + H (R1a2) product channels. Preliminary MESMER calculations using this surface and the various CCSD(T) calculated energies indicated that channels R1b and R1c are uncompetitive compared to channel R1a due to the emerged barriers TS3 and TS4, even at a temperature of 600 K. Bottom left panel: reduced surface containing only channel R1a that was used for MESMER fitting of experimental rate coefficient data and in which the postRC is set as a sink. Following fitting, the depth of the PRC was reduced to -7.7 kJ mol^{-1} and TS1 to -5.9 kJ mol^{-1}, and the surface used to calculate rate coefficients for the reaction over the temperature range T = 10 – 600 K. Bottom right panel: reduced surface containing only channel R1a but including the two possible exit channels R1a1 (C_2H_2 + CHO) and R1a2 (in which the CHO has dissociated into CO + H). This surface was used to determine the branching ratio between channels R1a1 and R1a2 over the temperature range T = 80 – 600 K.

Table S1. Optimized structures of the stationary points on the potential energy surface for C_2H + CH_2O shown in Figure 4 of the main text, calculated at the MP2/auc-cc-pVTZ level of theory.

Species	Structure	Species	Structure
C_2H	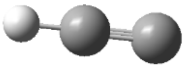	C_2H_2	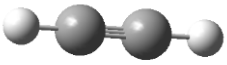
CH_2O	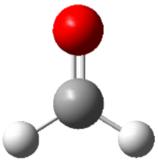	CHO	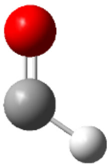
PRC	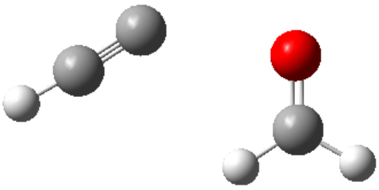	TS3	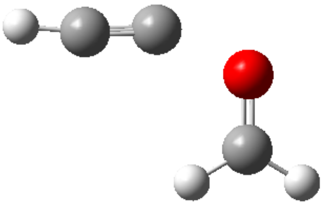
TS4	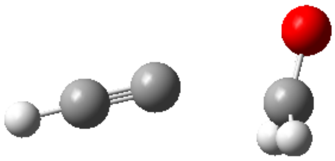	C-add	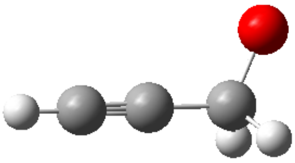
TS2	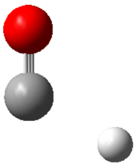	CO	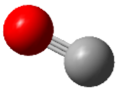
TS1	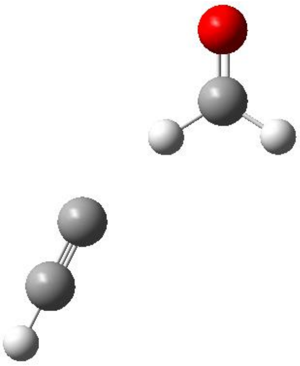	Post	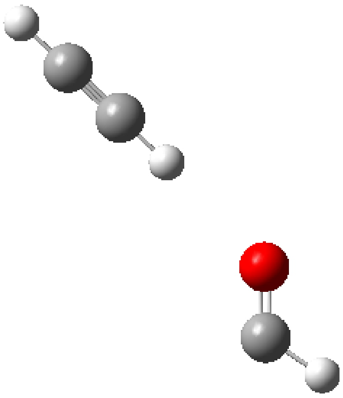
O-add	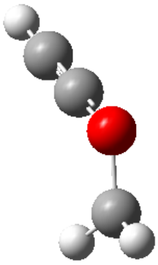		

Table S2. Optimized geometries of the stationary points on the potential energy surface for C_2H + CH_2O shown in Figure 4 of the main text, calculated at the MP2/auc-cc-pVTZ level of theory.

Species	Structure	Species	Structure
C_2H	C: 0.000000, 0.000000, 0.005886<br>H: 0.000000, 0.000000, 1.054119<br>C: 0.000000, 0.000000, -1.179433	C_2H_2	C: -0.126357, -2.318681, 0.000000<br>C: 1.085799, -2.318681, 0.000000<br>H: -1.188069, -2.318681, 0.000000<br>H: 2.147511, -2.318681, 0.000000
CH_2O	C: 0.189592, -0.296724, -0.282989<br>H: 0.694575, -1.203773, 0.081194<br>H: -0.833906, -0.122947, 0.081192<br>O: 0.733476, 0.472423, -1.046987	CHO	C: 2.099783, -0.898042, -0.415126<br>H: 3.111584, -1.320441, -0.623796<br>O: 1.651603, 0.051743, -0.960025
PRC	C: -2.301618, 0.284400, 0.030369<br>H: -1.931429, -0.750829, 0.037219<br>H: -2.015823, 0.932554, 0.871271<br>O: -2.997978, 0.695022, -0.869722<br>C: -3.417980, 3.019410, -0.213647<br>C: -3.075141, 3.657460, 0.714576<br>H: -2.789455, 4.267387, 1.533230	TS3	C: -2.324857, 0.470463, 0.099839<br>H: -2.012442, -0.574187, 0.006903<br>H: -2.005107, 1.064691, 0.965067<br>O: -3.024154, 0.969367, -0.754093<br>C: -3.409283, 2.804737, -0.323391<br>C: -3.004398, 3.357599, 0.656984<br>H: -2.749183, 4.012734, 1.451990
TS4	C: -1.435998, 0.585996, 0.055906<br>H: -0.975420, -0.386516, -0.184991<br>H: -0.786107, 1.466369, -0.080207<br>O: -2.530748, 0.670634, 0.537137<br>C: -2.131019, 0.774404, -2.020007<br>C: -2.298467, 0.857099, -3.179730<br>H: -2.436892, 0.930586, -4.229081	C-add	C: -1.413884, 0.610162, -0.409292<br>H: -0.920766, -0.332784, -0.141976<br>H: -0.740683, 1.398514, -0.050030<br>O: -2.570007, 0.690857, 0.335351<br>C: -1.588380, 0.705522, -1.863122<br>C: -1.716557, 0.782708, -3.065433<br>H: -1.839681, 0.851411, -4.117919
TS2	C: 1.859219, -0.794856, -0.367817<br>H: 3.434012, -1.499480, -0.653161<br>O: 1.569739, 0.127596, -0.977968	CO	C: 2.091427, -0.880314, -0.425302<br>O: 1.659995, 0.033987, -0.949846
TS1	C: 1.286758, -0.518038, -0.281380<br>H: 1.508856, -1.403996, 0.335639<br>H: 0.272335, -0.060176, -0.100883<br>O: 2.052193, -0.063803, -1.085814<br>C: -1.448361, 0.410341, 0.461077<br>H: -3.301062, 0.172879, 1.687896<br>C: -2.418522, 0.279559, 1.108875	Post	C: 2.447513, -1.039826, -0.489724<br>H: 3.339447, -0.860752, -1.134387<br>H: -0.658534, 0.082109, 0.296937<br>O: 1.469992, -0.372435, -0.504729<br>C: -1.636499, 0.326064, 0.635933<br>H: -3.728478, 0.847483, 1.361444<br>C: -2.751771, 0.604032, 1.022725
O-add	C: -1.946188, 0.478203, -0.041256<br>H: -1.954654, -0.586362, -0.184646<br>H: -1.176401, 1.009432, 0.493513<br>O: -3.221701, 1.008178, 0.009027<br>C: -3.295274, 2.274699, 0.332459<br>C: -3.403727, 3.442578, 0.632815<br>H: -3.508456, 4.465102, 0.891144		

Table S3. Rotational constants and unscaled vibrational frequencies of the stationary points on the potential energy surface for C_2H + CH_2O shown in Figure 4 of the main text, calculated at the MP2/aug-cc-pVTZ level of theory.

Species	Harmonic vibrational frequencies / cm^{-1}	Rotational constants / cm^{-1}
C_2H	821.14, 821.14, 2518.84, 3567.11	1.5485, 1.5485
CH_2O	1196.85, 1266.92, 1540.08, 1752.98, 2973.50, 3047.65	9.5460, 1.2845, 1.1321
PRC	74.77, 93.90, 138.49, 184.41, 223.67, 814.25, 832.62, 1233.58, 1269.61, 1538.14, 2187.05, 2518.63, 3011.81, 3067.72, 3561.78	0.9112, 0.1272, 0.1116
TS4	-497.06, 93.89, 106.02, 225.17, 446.84, 852.34, 860.38, 1201.45, 1271.75, 1547.07, 1775.67, 2562.99, 2952.01, 3025.83, 3566.05	1.2627, 0.1247, 0.1162
TS2	-1071.87, 472.21, 2080.74	9.3598, 1.4551, 1.2593
TS1	-219.42, 34.73, 48.69, 124.31, 174.28, 836.33, 841.61, 1202.75, 1237.54, 1496.99, 2091.15, 2384.27, 2545.11, 3008.78, 3561.24	2.3977, 0.0710, 0.0690
O-add	213.96, 241.82, 385.44, 540.72, 547.56, 591.56, 618.11, 954.81, 1195.78, 1260.37, 1479.13, 2211.06, 3205.36, 3372.05, 3504.33	1.6290, 0.1771, 0.1600
C_2H_2	601.32, 601.32, 753.86, 753.86, 1967.90, 3431.70, 3533.73	1.1688, 1.1688
CHO	1107.04, 1918.74, 2768.78	24.0674, 1.4817, 1.3957
TS3	-239.38, 74.61, 267.43, 279.68, 475.76, 750.53, 888.33, 1250.62, 1263.77, 1494.05, 2169.40, 2473.16, 3055.08, 3129.19, 3539.98	0.8873, 0.1811, 0.1504
C-add	205.24, 288.50, 500.92, 571.10, 667.75, 692.19, 936.87, 1069.47, 1163.07, 1357.17, 1416.46, 2347.42, 3028.82, 3061.71, 3501.90	1.3540, 0.1565, 0.1440
CO	2109.68	1.8954, 1.8954
Post	17.67, 39.26, 80.41, 91.66, 99.56, 622.73, 624.22, 784.29, 787.49, 1106.75, 1920.39, 1964.54, 2787.12, 3423.80, 3524.80	8.8366, 0.0559, 0.0556

Table S4. Optimized geometries of the stationary points on the potential energy surface for C_2H + CH_2O shown in Figure 4 of the main text, calculated at the M062X/6-311+G(3df,2p) level of theory.

Species	Structure	Species	Structure
C_2H	C: 0.000000, 0.000000, 0.000246<br>H: 0.000000, 0.000000, 1.064850<br>C: 0.000000, 0.000000, -1.196295	C_2H_2	H: -0.618408, 0.337238, 0.061392<br>C: -1.567084, 0.399399, 0.536083<br>H: -3.580582, 0.529955, 1.544784<br>C: -2.631970, 0.467950, 1.069947
CH_2O	C: 0.192308, -0.292922, -0.286849<br>H: 0.698190, -1.203169, 0.079125<br>H: -0.834456, -0.119291, 0.079052<br>O: 0.727695, 0.464361, -1.038919	CHO	C: 2.095721, -0.892042, -0.417885<br>H: 3.110941, -1.319036, -0.624613<br>O: 1.656308, 0.044338, -0.956449
PRC	C: 0.336803, -0.085345, 0.025749<br>H: -0.101578, -0.902438, -0.569769<br>H: -0.310096, 0.407093, 0.769153<br>O: 1.474316, 0.252825, -0.131259<br>C: 0.960576, 2.651925, 2.387254<br>H: 0.386795, 3.152877, 3.130220<br>C: 1.657037, 2.126740, 1.565064	TS3	C: 0.297326, -0.013224, 0.178913<br>H: 0.149874, -0.558799, -0.754626<br>H: -0.555090, 0.234257, 0.814180<br>O: 1.428418, 0.364869, 0.480133<br>C: 0.974320, 2.495954, 2.211369<br>H: 0.637745, 3.126905, 2.999931<br>C: 1.471259, 1.953716, 1.246512
TS4	C: 0.478059, 0.184533, -0.046062<br>H: 0.239590, -0.576465, 0.713326<br>H: -0.350013, 0.831037, -0.375979<br>O: 1.557869, 0.208480, -0.599803<br>C: 0.828179, 2.347213, 2.529730<br>H: 0.693733, 2.969070, 3.383197<br>C: 0.956435, 1.639809, 1.572002	C-add	C: -0.495895, 1.016443, -0.300511<br>H: -0.683034, 1.281388, 0.752275<br>H: -0.900802, -0.003378, -0.395849<br>O: 0.854651, 0.947396, -0.475363<br>C: -1.811201, 2.679011, -1.914884<br>H: -2.335779, 3.341759, -2.560033<br>C: -1.214216, 1.935678, -1.193632
TS2	C: 1.784027, -0.762046, -0.353496<br>H: 3.527028, -1.546516, -0.665488<br>O: 1.551915, 0.141822, -0.979964	CO	C: 2.086986, -0.873428, -0.428591<br>O: 1.665043, 0.025724, -0.945743
TS1	C: 1.352516, -0.525616, -0.310760<br>H: 1.612967, -1.414973, 0.288832<br>H: 0.335592, -0.090647, -0.110591<br>O: 2.086291, -0.051486, -1.115030<br>C: -1.487148, 0.339660, 0.536647<br>H: -3.432873, 0.265342, 1.687549<br>C: -2.515148, 0.294487, 1.148763	Post	C: 2.330598, -1.048449, -0.491616<br>H: 3.348524, -1.008380, -0.954989<br>H: -0.666244, 0.214282, 0.170825<br>O: 1.510683, -0.229564, -0.631330<br>C: -1.634912, 0.377373, 0.584682<br>H: -3.686605, 0.721619, 1.462192<br>C: -2.720374, 0.559793, 1.048436
O-add	C: 0.164657, -0.200855, 0.483542<br>H: 0.377606, -1.139769, 0.004198<br>H: -0.509044, -0.084958, 1.317790<br>O: 1.208036, 0.687923, 0.394674<br>C: 1.049234, 2.817197, 1.682878<br>H: 1.003513, 3.730231, 2.222291<br>C: 1.109852, 1.793908, 1.071039		

Table S5. Rotational constants and unscaled vibrational frequencies of the stationary points on the potential energy surface for C_2H + CH_2O shown in Figure 4 of the main text, calculated at the M062X/6-311+G(3df,2p) level of theory.

Species	Harmonic vibrational frequencies / cm^{-1}	Rotational constants / cm^{-1}
C_2H	528.43, 528.43, 2134.57, 3457.64	1.4965, 1.4965
CH_2O	1214.63, 1274.80, 1540.12, 1879.52, 2968.19, 3038.67	9.4936, 1.3196, 1.1586
PRC	64.39, 100.15, 165.68, 194.63, 236.84, 616.92, 647.15, 1222.18, 1273.31, 1526.46, 1864.69, 2112.98, 2993.80, 3076.15, 3456.28	0.9573, 0.1176, 0.1048
TS4	-388.00, 79.35, 92.98, 208.58, 454.64, 620.83, 635.66, 1126.24, 1249.98, 1509.93, 1692.60, 2111.72, 2987.60, 3064.02, 3436.71	1.3129, 0.1186, 0.1113
TS2	-634.57, 343.07, 2231.21	7.4398, 1.4702, 1.2276
TS1	-160.49, 40.91, 82.40, 114.56, 207.35, 610.61, 635.19, 1198.28, 1242.29, 1495.14, 1886.92, 2110.21, 2510.71, 2995.19, 3451.19	2.5476, 0.0666, 0.0649
O-add	234.01, 259.20, 479.41, 546.37, 602.93, 608.66, 692.06, 977.44, 1197.28, 1291.66, 1465.20, 2316.73, 3173.63, 3330.85, 3485.31	1.7020, 0.1778, 0.1612
C_2H_2	699.94, 699.94, 780.81, 785.70, 2102.16, 3407.16, 3514.00	1.1963, 1.1963
CHO	1115.68, 2013.15, 2728.98	23.9997, 1.5198, 1.4293
TS3	-739.95, 107.74, 159.64, 409.55, 492.55, 638.55, 688.45, 1085.12, 1237.72, 1429.53, 1571.57, 1993.42, 3056.94, 3177.05, 3442.34	1.1235, 0.1646, 0.1471
C-add	213.78, 308.38, 584.38, 608.48, 715.21, 737.79, 930.22, 1083.74, 1159.80, 1341.72, 1394.76, 2260.97, 2979.37, 2996.45, 3462.86	1.3767, 0.1578, 0.1453
CO	2271.87	1.9608, 1.9608
Post	55.57, 69.88, 76.47, 91.23, 189.21, 718.92, 725.94, 813.95, 816.97, 1108.48, 1999.90, 2091.28, 2782.77, 3384.52, 3497.54	4.3326, 0.0573, 0.0566

Table S6. Comparison of the relative energies of the stationary points on the potential energy surface for $C_2H + CH_2O$ shown in Figure 4 of the main text calculated at various levels of theory. Also included are literature values taken from the Active ThermoChemical Tables (ATcT).[3, 4] All energies are given in kJ mol^{-1} and include zero-point energy.

Species	This work				Dong *et. al.*[5]								ATcT[m]
	MP2[a]	CCSD(T) //MP2[b]	M062X[c]	CCSD(T) //M062X[d]	B3LYP[e]	CCSD(T) //B3LYP[f]	CCSD(T) //B3LYP[g]	MP2[h]	CCSD(T) //MP2[i]	QCSID[j]	CCSD(T) //QCSID[k]	G3MP[l]	
PRC	-5.2	-4.4	-13.5	-5.4	-18.4	-5.4	-2.9	-5.0	-3.35	-6.3	-6.28	-8.4	
TS1	3.1	-2.6	-5.9	-2.0				7.1	-2.51	1.7	-2.93	-14.6	
Post RC	-255.4	-207.5	-190.1	-193.0		-188.7							
C_2H_2 + CHO	-250.0	-203.5	-189.2	-194.1		-186.2							-188.1
TS2	-174.1	-130.0	-111.9	-119.1		-129.7							
C_2H_2 + CO + H	-201.6	-143.5	-129.8	-133.8		-144.8							-127.3
TS3	0.7	12.8	26.7	31.24	5.9	36.8	33.5	14.6	22.18	55.2	36.40	12.6	
O-add	-200.3	-156.1	-162.6	-143.4		-124.3							
TS4	42.3	118.9	6.3	108.4	3.3	9.6	8.4	51.5	15.06	20.9	12.13	8.8	
C-add	-215.7	-186.9	-186.0	-176.4		-164.8							

Levels of theory used in calculation:
This work:
[a] MP2/aug-cc-pVTZ
[b] CCSD(T) energies extrapolated to the complete basis set limit using aug-cc-pVXZ (X = 2, 3, 4) and the MP2/aug-cc-pVTZ optimized structures.
[c] M062X/6-311+G(3*df*,2*p*)
[d] CCSD(T) energies extrapolated to the complete basis set limit using aug-cc-pVXZ (X = 2, 3, 4) and the M062X/6-311+G(3*df*,2*p*) optimized structures.
Dong *et. al.*:[5]
[e] B3LYP/6-311G(*d*,*p*)
[f] CCSD(T)/6-311G(2*d*,*p*)//B3LYP/6-311G(*d*,*p*)
[g] CCSD(T)/6-311+G(3*df*,2*p*)//B3LYP/6-311G(*d*,*p*)
[h] MP2/6-311G(*d*,*p*)
[i] CCSD(T)6-311+G(3*df*,2*p*)//MP2/6-311G(*d*,*p*)
[j] QCISD/6-311G(*d*,*p*)
[k] CCSD(T)6-311+G(3*df*,2*p*)//QCISD/6-311G(*d*,*p*)
[l] G3MP2
[m] Reactions enthalpies (ΔH_f 0K) taken from the Active Thermochemical Tables (ATcT).[3, 4]

Table S7. Experimental *vs* calculated rate coefficients for C_2H + CH_2O (R1) following fitting.

T / K	Rate coefficient, k_1 / 10^{-10} cm^3 $molecule^{-1}$ s^{-1}		% Difference
	Experiment	MESMER	
37 ± 3	1.2 ± 0.1	1.22	4 %
37 ± 3	1.4 ± 0.2	1.22	13 %
44 ± 4	2.0 ± 0.4	1.14	44 %
51 ± 4	0.89 ± 0.06	1.08	21 %
61 ± 6	1.4 ± 0.1	0.99	31 %
67 ± 2	0.91 ± 0.09	0.95	4 %
83 ± 3	0.72 ± 0.09	0.85	18 %
83 ± 3	1.0 ± 0.07	0.85	17 %
93 ± 7	0.73 ± 0.10	0.80	11 %
93 ± 7	0.79 ± 0.10	0.80	1 %
308 ± 5	0.47 ±0.05	0.51	8 %
603 ± 5	0.60 ± 0.06	0.56	6 %
603 ± 5	0.56 ± 0.06	0.56	1 %
			Average = 14 %

Table S8. BRs for channel R1a2 as calculated by MESMER and fit with a single exponential and extrapolated down to 0 K. The BRs have been applied to the MESMER calculated rate coefficients in order to give temperature dependent channel specific rate coefficients (for channels R1a1, C_2H_2 + CHO, and R1a2, C_2H_2 + CO + H).

T / K	% Branching ratio for channel R1a2		Rate coefficient (cm^3 molecule^{-1} s^{-1})	
	MESMER	Exponential fit	k_{1a1}	k_{1a1}
600	58.5	58.6	2.34E-11	3.31E-11
550		57.4	2.34E-11	3.14E-11
500	56.3	56.3	2.33E-11	3E-11
450		55.2	2.32E-11	2.87E-11
400	54.4	54.3	2.33E-11	2.77E-11
350		53.5	2.35E-11	2.7E-11
300	52.7	52.7	2.4E-11	2.68E-11
275	52.3	52.3	2.45E-11	2.69E-11
250	51.9	52.0	2.51E-11	2.72E-11
225	51.6	51.6	2.6E-11	2.78E-11
200	51.3	51.3	2.72E-11	2.87E-11
175	51	51.0	2.88E-11	3.01E-11
150	50.7	50.7	3.11E-11	3.2E-11
125	50.4	50.5	3.42E-11	3.49E-11
100	50.2	50.2	3.87E-11	3.9E-11
90	50.1	50.1	4.1E-11	4.12E-11
80	50.1	50.0	4.37E-11	4.37E-11
70		49.9	4.68E-11	4.66E-11
60		49.8	5.05E-11	5.01E-11
50		49.7	5.49E-11	5.43E-11
40		49.6	6.04E-11	5.94E-11
30		49.5	6.76E-11	6.63E-11
30		49.5	6.76E-11	6.63E-11
25		49.5	7.27E-11	7.11E-11
20		49.4	8.02E-11	7.83E-11
15		49.4	9.41E-11	9.17E-11
10		49.3	1.35E-10	1.31E-10

References

(1) Smith, C. A.; Pope, F. D.; Cronin, B.; Parkes, C. B.; Orr-Ewing, A. J. Absorption cross sections of formaldehyde at wavelengths from 300 to 340 nm at 294 and 245 K. *J. Phys. Chem. A* **2006**, *110* (41), 11645-11653. DOI: 10.1021/jp063713y.

(2) West, N. A.; Millar, T. J.; Van de Sande, M.; Rutter, E.; Blitz, M. A.; Decin, L.; Heard, D. E. Measurements of Low Temperature Rate Coefficients for the Reaction of CH with CH_2O and Application to Dark Cloud and AGB Stellar Wind Models. *Astrophys. J.* **2019**, *885* (2), 134. DOI: 10.3847/1538-4357/ab480e.

(3) *Active Thermochemical Tables*. Argonne National Laboratory, 2024. https://atct.anl.gov/ (accessed 04/09/2024).

(4) Ruscic, B.; Pinzon, R. E.; Morton, M. L.; von Laszevski, G.; Bittner, S. J.; Nijsure, S. G.; Amin, K. A.; Minkoff, M.; Wagner, A. F. Introduction to Active Thermochemical Tables: Several “Key” Enthalpies of Formation Revisited. *J. Phys. Chem. A* **2004**, *108* (45), 9979-9997. DOI: 10.1021/jp047912y.

(5) Dong, H.; Ding, Y. H.; Sun, C. C. C_2H + H_2CO: A new route for formaldehyde removal. *J. Chem. Phys.* **2005**, *122* (20). DOI: 10.1063/1.1903945.
