# Supplementary material for: Experimental and Theoretical Investigation of the Reaction of C2H with Formaldehyde (CH2O) at Very Low Temperatures and Application to Astrochemical Models
Source: ACS Earth Space Chem. 2024 Nov 20;8(12):2428–41. doi: 10.1021/acsearthspacechem.4c00188 (PMC11664655; doi:10.1021/acsearthspacechem.4c00188)
Supplement: Supplementary file 2 — sp4c00188_si_002.pdf [file sp4c00188_si_002.pdf]

```
<?xml version="1.0" encoding="utf-8" ?>
<?xml-stylesheet type='text/xsl' href='../../mesmer2.xsl' media='other'?>
<?xml-stylesheet type='text/xsl' href='../../mesmer1.xsl' media='screen'?>
<me:mesmer xmlns="http://www.xml-cml.org/schema" xmlns:me=
"http://www.chem.leeds.ac.uk/mesmer" xmlns:xsi=
"http://www.w3.org/2001/XMLSchema-instance">

  <me:title>C2H+CH2O mp2-ccsd(t)  020_mesmer_in</me:title>

  <moleculeList convention="">

    <molecule id="C2H">
      <atomArray>
        <atom id="a1" elementType="C" x3="0.000000" y3="0.000000" z3="0.005886" />
        <atom id="a2" elementType="H" x3="0.000000" y3="0.000000" z3="1.054119" />
        <atom id="a3" elementType="C" x3="0.000000" y3="0.000000" z3="-1.179433" />
      </atomArray>
      <bondArray>
        <bond atomRefs2="a1 a2" order="3" />
        <bond atomRefs2="a1 a3" order="1" />
      </bondArray>
      <propertyList>
        <property dictRef="me:ZPE">
          <scalar units="kJ/mol">0</scalar>
        </property>
        <property dictRef="me:frequenciesScaleFactor">
          <scalar>0.953</scalar>
          <!-- <scalar>0.956 </scalar> !For m062x-->
        </property>
        <property dictRef="me:vibFreqs">
          <array units="cm-1">821.141 821.141 2518.8384 3567.1075</array>
        </property>
        <property dictRef="me:rotConsts">
          <array units="cm-1">1.548528716 1.548528716</array>
        </property>
        <property dictRef="me:symmetryNumber">
          <scalar>1</scalar>
        </property>
        <property dictRef="me:MW">
          <scalar units="amu">25.03</scalar>
        </property>
        <property dictRef="me:spinMultiplicity">
          <scalar>2</scalar>
        </property>
        <property dictRef="me:epsilon">
          <scalar>200</scalar>
        </property>
        <property dictRef="me:sigma">
          <scalar>4</scalar>
        </property>
      </propertyList>
      <me:DOSCMethod xsi:type="QMRotors" />
      <me:energyTransferModel xsi:type="me:ExponentialDown">
        <me:deltaEDown bathGas="N2" units="cm-1">250</me:deltaEDown>
        <me:deltaEDownTExponent bathGas="N2" referenceTemperature="298">0.25
        </me:deltaEDownTExponent>
        <me:deltaEDown bathGas="Ar" units="cm-1">200</me:deltaEDown>
        <me:deltaEDownTExponent bathGas="Ar" referenceTemperature="298">0.5
        </me:deltaEDownTExponent>
        <me:deltaEDown bathGas="He" units="cm-1">100</me:deltaEDown>
        <me:deltaEDownTExponent bathGas="He" referenceTemperature="298">1.0
        </me:deltaEDownTExponent>
      </me:energyTransferModel>
      <me:DistributionCalcMethod default="true" name="Boltzmann" />
      <me:densityOfStatesList calculated="20231201_135454">
        <me:description>Rovibronic partition function calculation at various
        temperatures. qtot : product of QM partition functions for vibrations (1-D
        harmonic oscillator) and classical partition functions for rotations.  sumc :
        cell based partition function. sumg : grain based partition function
        </me:description>
        <me:densityOfStates>
          <me:T>200</me:T>
```

```
64              <me:qtot>180.831</me:qtot>
65              <me:sumc>180.972</me:sumc>
66              <me:sumg>180.743</me:sumg>
67            </me:densityOfStates>
68            <me:densityOfStates>
69              <me:T>300</me:T>
70              <me:qtot>282.391</me:qtot>
71              <me:sumc>282.495</me:sumc>
72              <me:sumg>282.362</me:sumg>
73            </me:densityOfStates>
74            <me:densityOfStates>
75              <me:T>400</me:T>
76              <me:qtot>406.374</me:qtot>
77              <me:sumc>406.416</me:sumc>
78              <me:sumg>406.325</me:sumg>
79            </me:densityOfStates>
80          </me:densityOfStatesList>
81        </molecule>
82
83        <molecule id="CH2O">
84          <atomArray>
85            <atom id="a1" elementType="C" x3="0.189592" y3="-0.296724" z3="-0.282989" />
86            <atom id="a2" elementType="H" x3="0.694575" y3="-1.203773" z3="0.081194" />
87            <atom id="a3" elementType="H" x3="-0.833906" y3="-0.122947" z3="0.081192" />
88            <atom id="a4" elementType="O" x3="0.733476" y3="0.472423" z3="-1.046987" />
89          </atomArray>
90          <bondArray>
91            <bond atomRefs2="a1 a2" order="1" />
92            <bond atomRefs2="a1 a3" order="1" />
93            <bond atomRefs2="a1 a4" order="2" />
94          </bondArray>
95          <propertyList>
96            <property dictRef="me:ZPE">
97              <scalar units="kJ/mol">0</scalar>
98            </property>
99            <property dictRef="me:frequenciesScaleFactor">
100             <scalar>0.953</scalar>
101           </property>
102           <property dictRef="me:vibFreqs">
103             <array units="cm-1">1196.8503 1266.9194 1540.0817 1752.9846 2973.4988
              3047.6474</array>
104           </property>
105           <property dictRef="me:rotConsts">
106             <array units="cm-1">9.546036678 1.284465268 1.132131549</array>
107           </property>
108           <property dictRef="me:symmetryNumber">
109             <scalar>2</scalar>
110           </property>
111           <property dictRef="me:MW">
112             <scalar units="amu">30.03</scalar>
113           </property>
114           <property dictRef="me:spinMultiplicity">
115             <scalar>1</scalar>
116           </property>
117           <property dictRef="me:epsilon">
118             <scalar>300</scalar>
119           </property>
120           <property dictRef="me:sigma">
121             <scalar>4.2</scalar>
122           </property>
123         </propertyList>
124         <me:DOSCMethod xsi:type="QMRotors" />
125         <me:energyTransferModel xsi:type="me:ExponentialDown">
126           <me:deltaEDown bathGas="N2" units="cm-1">250</me:deltaEDown>
127           <me:deltaEDownTExponent bathGas="N2" referenceTemperature="298">0.25
              </me:deltaEDownTExponent>
128           <me:deltaEDown bathGas="Ar" units="cm-1">200</me:deltaEDown>
129           <me:deltaEDownTExponent bathGas="Ar" referenceTemperature="298">0.5
              </me:deltaEDownTExponent>
130           <me:deltaEDown bathGas="He" units="cm-1">100</me:deltaEDown>
131           <me:deltaEDownTExponent bathGas="He" referenceTemperature="298">1.0
              </me:deltaEDownTExponent>
```

```
      </me:energyTransferModel>
    </molecule>

    <molecule id="PRC">
      <atomArray>
        <atom id="a1" elementType="C" x3="-2.301618" y3="0.284400" z3="0.030369" />
        <atom id="a2" elementType="H" x3="-1.931429" y3="-0.750829" z3="0.037219" />
        <atom id="a3" elementType="H" x3="-2.015823" y3="0.932554" z3="0.871271" />
        <atom id="a4" elementType="O" x3="-2.997978" y3="0.695022" z3="-0.869722" />
        <atom id="a5" elementType="C" x3="-3.417980" y3="3.019410" z3="-0.213647" />
        <atom id="a6" elementType="C" x3="-3.075141" y3="3.657460" z3="0.714576" />
        <atom id="a7" elementType="H" x3="-2.789455" y3="4.267387" z3="1.533230" />
      </atomArray>
      <bondArray>
        <bond atomRefs2="a1 a2" order="1" />
        <bond atomRefs2="a1 a3" order="1" />
        <bond atomRefs2="a1 a4" order="2" />
        <bond atomRefs2="a4 a5" order="1" />
        <bond atomRefs2="a5 a6" order="3" />
        <bond atomRefs2="a6 a7" order="1" />
      </bondArray>
      <propertyList>
        <property dictRef="me:ZPE">
          <!-- <scalar units="kJ/mol">-4.3725 </scalar> -->
          <scalar units="kJ/mol">-7.7</scalar>
          <!-- <scalar units="kJ/mol" lower="-10.0" upper="-0.5"
          stepsize="0.10">-7.58 </scalar> -->
        </property>
        <property dictRef="me:frequenciesScaleFactor">
          <scalar>0.953</scalar>
        </property>
        <property dictRef="me:vibFreqs">
          <array units="cm-1">74.7737 93.9041 138.4934 184.4052 223.6679 814.2472
          832.6245 1233.5782 1269.6078 1538.1447 2187.0453 2518.6259 3011.8097
          3067.7177 3561.7834</array>
        </property>
        <property dictRef="me:rotConsts">
          <array units="cm-1">0.911240736 0.127210338 0.111626891</array>
        </property>
        <property dictRef="me:symmetryNumber">
          <scalar>1</scalar>
        </property>
        <property dictRef="me:MW">
          <scalar units="amu">55.06</scalar>
        </property>
        <property dictRef="me:spinMultiplicity">
          <scalar>2</scalar>
        </property>
        <property dictRef="me:epsilon">
          <scalar>350</scalar>
        </property>
        <property dictRef="me:sigma">
          <scalar>5</scalar>
        </property>
      </propertyList>
      <me:DOSCMethod xsi:type="QMRotors" />
      <me:energyTransferModel xsi:type="me:ExponentialDown">
        <me:deltaEDown bathGas="N2" units="cm-1">150</me:deltaEDown>
        <me:deltaEDownTExponent bathGas="N2" referenceTemperature="298">0.0
        </me:deltaEDownTExponent>
        <me:deltaEDown bathGas="Ar" units="cm-1">100</me:deltaEDown>
        <me:deltaEDownTExponent bathGas="Ar" referenceTemperature="298">0.0
        </me:deltaEDownTExponent>
        <me:deltaEDown bathGas="He" units="cm-1">50</me:deltaEDown>
        <me:deltaEDownTExponent bathGas="He" referenceTemperature="298">0.0
        </me:deltaEDownTExponent>
      </me:energyTransferModel>
      <me:DistributionCalcMethod default="true" name="Boltzmann" />
      <me:densityOfStatesList calculated="20231201_135454">
        <me:description>Rovibronic partition function calculation at various
        temperatures. qtot : product of QM partition functions for vibrations (1-D
        harmonic oscillator) and classical partition functions for rotations.  sumc :
```

```
          cell based partition function. sumg : grain based partition function
          </me:description>
          <me:densityOfStates>
            <me:T>200</me:T>
            <me:qtot>783475</me:qtot>
            <me:sumc>784042</me:sumc>
            <me:sumg>782527</me:sumg>
          </me:densityOfStates>
          <me:densityOfStates>
            <me:T>300</me:T>
            <me:qtot>5.73615e+06</me:qtot>
            <me:sumc>5.75067e+06</me:sumc>
            <me:sumg>5.74572e+06</me:sumg>
          </me:densityOfStates>
          <me:densityOfStates>
            <me:T>400</me:T>
            <me:qtot>2.86217e+07</me:qtot>
            <me:sumc>2.87209e+07</me:sumc>
            <me:sumg>2.8707e+07</me:sumg>
          </me:densityOfStates>
        </me:densityOfStatesList>
      </molecule>

      <molecule id="TS">
        <atomArray>
          <atom id="a1" elementType="C" x3="1.286758" y3="-0.518038" z3="-0.281380" />
          <atom id="a2" elementType="H" x3="1.508856" y3="-1.403996" z3="0.335639" />
          <atom id="a3" elementType="H" x3="0.272335" y3="-0.060176" z3="-0.100883" />
          <atom id="a4" elementType="O" x3="2.052193" y3="-0.063803" z3="-1.085814" />
          <atom id="a5" elementType="C" x3="-1.448361" y3="0.410341" z3="0.461077" />
          <atom id="a6" elementType="H" x3="-3.301062" y3="0.172879" z3="1.687896" />
          <atom id="a7" elementType="C" x3="-2.418522" y3="0.279559" z3="1.108875" />
        </atomArray>
        <bondArray>
          <bond atomRefs2="a1 a2" order="1" />
          <bond atomRefs2="a1 a3" order="1" />
          <bond atomRefs2="a1 a4" order="2" />
          <bond atomRefs2="a3 a5" order="1" />
          <bond atomRefs2="a5 a7" order="3" />
          <bond atomRefs2="a7 a6" order="1" />
        </bondArray>
        <propertyList>
          <property dictRef="me:ZPE">
            <scalar units="kJ/mol">-5.90 </scalar>
            <!-- <scalar units="kJ/mol" lower="-7.5" upper="2.5"
            stepsize="0.05">-2.6</scalar> -->
          </property>
          <property dictRef="me:frequenciesScaleFactor">
            <scalar>0.953</scalar>
          </property>
          <property dictRef="me:vibFreqs">
            <array units="cm-1">34.7288 48.6867 124.3136 174.2798 836.327 841.61
            1202.7542 1237.5432 1496.9883 2091.1505 2384.2691 2545.1141 3008.7828
            3561.2372</array>
          </property>
          <property dictRef="me:rotConsts">
            <array units="cm-1">2.397679731 0.071043482 0.068999067</array>
          </property>
          <property title="ImaginaryFrequency" dictRef="me:imFreqs">
            <scalar units="cm-1">219.4242</scalar>
            <!-- <scalar units="cm-1" lower="50" upper="2000" stepsize="5">219.4242
            </scalar> -->
          </property>
          <property dictRef="me:symmetryNumber">
            <scalar>1</scalar>
          </property>
          <property dictRef="me:MW">
            <scalar units="amu">55.06</scalar>
          </property>
          <property dictRef="me:spinMultiplicity">
            <scalar>2</scalar>
          </property>
```

```
262           <property dictRef="me:epsilon">
263             <scalar>350</scalar>
264           </property>
265           <property dictRef="me:sigma">
266             <scalar>5</scalar>
267           </property>
268         </propertyList>
269         <me:DOSCMethod xsi:type="QMRotors" />
270         <me:energyTransferModel xsi:type="me:ExponentialDown">
271           <me:deltaEDown bathGas="N2" units="cm-1">250</me:deltaEDown>
272           <me:deltaEDownTExponent bathGas="N2" referenceTemperature="298">0.25
              </me:deltaEDownTExponent>
273           <me:deltaEDown bathGas="Ar" units="cm-1">200</me:deltaEDown>
274           <me:deltaEDownTExponent bathGas="Ar" referenceTemperature="298">0.5
              </me:deltaEDownTExponent>
275           <me:deltaEDown bathGas="He" units="cm-1">100</me:deltaEDown>
276           <me:deltaEDownTExponent bathGas="He" referenceTemperature="298">1.0
              </me:deltaEDownTExponent>
277         </me:energyTransferModel>
278         <me:densityOfStatesList calculated="20231201_135454">
279           <me:description>Rovibronic partition function calculation at various
              temperatures. qtot : product of QM partition functions for vibrations (1-D
              harmonic oscillator) and classical partition functions for rotations.  sumc :
              cell based partition function. sumg : grain based partition function
              </me:description>
280           <me:densityOfStates>
281             <me:T>200</me:T>
282             <me:qtot>2.24408e+06</me:qtot>
283             <me:sumc>2.26447e+06</me:sumc>
284             <me:sumg>2.2601e+06</me:sumg>
285           </me:densityOfStates>
286           <me:densityOfStates>
287             <me:T>300</me:T>
288             <me:qtot>1.48874e+07</me:qtot>
289             <me:sumc>1.50564e+07</me:sumc>
290             <me:sumg>1.50434e+07</me:sumg>
291           </me:densityOfStates>
292           <me:densityOfStates>
293             <me:T>400</me:T>
294             <me:qtot>6.56781e+07</me:qtot>
295             <me:sumc>6.65013e+07</me:sumc>
296             <me:sumg>6.64691e+07</me:sumg>
297           </me:densityOfStates>
298         </me:densityOfStatesList>
299       </molecule>
300
301       <molecule id="PostRC">
302         <atomArray>
303           <atom id="a1" elementType="C" x3="2.447513" y3="-1.039826" z3="-0.489724" />
304           <atom id="a2" elementType="H" x3="3.339447" y3="-0.860752" z3="-1.134387" />
305           <atom id="a3" elementType="H" x3="-0.658534" y3="0.082109" z3="0.296937" />
306           <atom id="a4" elementType="O" x3="1.469992" y3="-0.372435" z3="-0.504729" />
307           <atom id="a5" elementType="C" x3="-1.636499" y3="0.326064" z3="0.635933" />
308           <atom id="a6" elementType="C" x3="-3.728478" y3="0.847483" z3="1.361444" />
309           <atom id="a7" elementType="H" x3="-2.751771" y3="0.604032" z3="1.022725" />
310         </atomArray>
311         <bondArray>
312           <bond atomRefs2="a1 a2" order="1" />
313           <bond atomRefs2="a4 a3" order="1" />
314           <bond atomRefs2="a1 a4" order="2" />
315           <bond atomRefs2="a7 a5" order="3" />
316           <bond atomRefs2="a5 a3" order="1" />
317           <bond atomRefs2="a6 a7" order="1" />
318         </bondArray>
319         <propertyList>
320           <property dictRef="me:ZPE">
321             <scalar units="kJ/mol">-207.5441</scalar>
322             <!-- <scalar units="kJ/mol" lower="-10.0" upper="-0.5"
              stepsize="0.10">-207.5 </scalar> -->
323           </property>
324           <property dictRef="me:frequenciesScaleFactor">
325             <scalar>0.953</scalar>
```

```
326           </property>
327           <property dictRef="me:vibFreqs">
328             <array units="cm-1">17.6687 39.2596 80.4102 91.6597 99.5551 622.7305
              624.2228 784.2895 787.4926 1106.7498 1920.3923 1964.5391 2787.1164
              3423.7966 3524.7977</array>
329           </property>
330           <property dictRef="me:rotConsts">
331             <array units="cm-1">8.836647585 0.055922354 0.055570778</array>
332           </property>
333           <property dictRef="me:symmetryNumber">
334             <scalar>1</scalar>
335           </property>
336           <property dictRef="me:MW">
337             <scalar units="amu">55.02</scalar>
338           </property>
339           <property dictRef="me:spinMultiplicity">
340             <scalar>2</scalar>
341           </property>
342           <property dictRef="me:epsilon">
343             <scalar>350</scalar>
344           </property>
345           <property dictRef="me:sigma">
346             <scalar>5</scalar>
347           </property>
348         </propertyList>
349         <me:DOSCMethod xsi:type="QMRotors" />
350         <me:energyTransferModel xsi:type="me:ExponentialDown">
351           <me:deltaEDown bathGas="N2" units="cm-1">150</me:deltaEDown>
352           <me:deltaEDownTExponent bathGas="N2" referenceTemperature="298">0.0
              </me:deltaEDownTExponent>
353           <me:deltaEDown bathGas="Ar" units="cm-1">100</me:deltaEDown>
354           <me:deltaEDownTExponent bathGas="Ar" referenceTemperature="298">0.0
              </me:deltaEDownTExponent>
355           <me:deltaEDown bathGas="He" units="cm-1">50</me:deltaEDown>
356           <me:deltaEDownTExponent bathGas="He" referenceTemperature="298">0.0
              </me:deltaEDownTExponent>
357         </me:energyTransferModel>
358         <me:DistributionCalcMethod default="true" name="Boltzmann" />
359         <me:densityOfStatesList calculated="20231201_135454">
360           <me:description>Rovibronic partition function calculation at various
              temperatures. qtot : product of QM partition functions for vibrations (1-D
              harmonic oscillator) and classical partition functions for rotations.  sumc :
              cell based partition function. sumg : grain based partition function
              </me:description>
361           <me:densityOfStates>
362             <me:T>200</me:T>
363             <me:qtot>1.38273e+07</me:qtot>
364             <me:sumc>1.37685e+07</me:sumc>
365             <me:sumg>1.37419e+07</me:sumg>
366           </me:densityOfStates>
367           <me:densityOfStates>
368             <me:T>300</me:T>
369             <me:qtot>1.57532e+08</me:qtot>
370             <me:sumc>1.57081e+08</me:sumc>
371             <me:sumg>1.56946e+08</me:sumg>
372           </me:densityOfStates>
373           <me:densityOfStates>
374             <me:T>400</me:T>
375             <me:qtot>1.08222e+09</me:qtot>
376             <me:sumc>1.07999e+09</me:sumc>
377             <me:sumg>1.07947e+09</me:sumg>
378           </me:densityOfStates>
379         </me:densityOfStatesList>
380       </molecule>
381
382       <molecule id="CHO">
383         <atomArray>
384           <atom id="a1" elementType="C" x3="2.099783" y3="-0.898042" z3="-0.415126" />
385           <atom id="a2" elementType="H" x3="3.111584" y3="-1.320441" z3="-0.623796" />
386           <atom id="a3" elementType="O" x3="1.651603" y3="0.051743" z3="-0.960025" />
387         </atomArray>
388         <bondArray>
```

```
          <bond atomRefs2="a1 a2" order="1" />
          <bond atomRefs2="a1 a3" order="2" />
        </bondArray>
        <propertyList>
          <property dictRef="me:ZPE">
            <scalar units="kJ/mol">-203.5017</scalar>
          </property>
          <property dictRef="me:frequenciesScaleFactor">
            <scalar>0.953</scalar>
          </property>
          <property dictRef="me:vibFreqs">
            <array units="cm-1">1107.0426 1918.7407 2768.7847</array>
          </property>
          <property dictRef="me:rotConsts">
            <array units="cm-1">24.06743868 1.4816757 1.395748588</array>
          </property>
          <property dictRef="me:symmetryNumber">
            <scalar>1</scalar>
          </property>
          <property dictRef="me:MW">
            <scalar units="amu">29.02</scalar>
          </property>
          <property dictRef="me:spinMultiplicity">
            <scalar>2</scalar>
          </property>
          <property dictRef="me:epsilon">
            <scalar>224</scalar>
          </property>
          <property dictRef="me:sigma">
            <scalar>4.13</scalar>
          </property>
        </propertyList>
        <me:DOSCMethod xsi:type="QMRotors" />
        <me:energyTransferModel xsi:type="me:ExponentialDown">
          <me:deltaEDown bathGas="N2" units="cm-1">250</me:deltaEDown>
          <me:deltaEDownTExponent bathGas="N2" referenceTemperature="298">0.25
          </me:deltaEDownTExponent>
          <me:deltaEDown bathGas="Ar" units="cm-1">200</me:deltaEDown>
          <me:deltaEDownTExponent bathGas="Ar" referenceTemperature="298">0.5
          </me:deltaEDownTExponent>
          <me:deltaEDown bathGas="He" units="cm-1">100</me:deltaEDown>
          <me:deltaEDownTExponent bathGas="He" referenceTemperature="298">1.0
          </me:deltaEDownTExponent>
        </me:energyTransferModel>
        <me:DistributionCalcMethod default="true" name="Boltzmann" />
        <me:densityOfStatesList calculated="20231201_135454">
          <me:description>Rovibronic partition function calculation at various
          temperatures. qtot : product of QM partition functions for vibrations (1-D
          harmonic oscillator) and classical partition functions for rotations.  sumc :
          cell based partition function. sumg : grain based partition function
          </me:description>
          <me:densityOfStates>
            <me:T>200</me:T>
            <me:qtot>823.922</me:qtot>
            <me:sumc>823.515</me:sumc>
            <me:sumg>822.296</me:sumg>
          </me:densityOfStates>
          <me:densityOfStates>
            <me:T>300</me:T>
            <me:qtot>1522.78</me:qtot>
            <me:sumc>1521.97</me:sumc>
            <me:sumg>1521.02</me:sumg>
          </me:densityOfStates>
          <me:densityOfStates>
            <me:T>400</me:T>
            <me:qtot>2386.3</me:qtot>
            <me:sumc>2385.02</me:sumc>
            <me:sumg>2384.2</me:sumg>
          </me:densityOfStates>
        </me:densityOfStatesList>
      </molecule>
```

```
<molecule id="C2H2">
  <atomArray>
    <atom id="a1" elementType="C" x3="-0.126357" y3="-2.318681" z3="0.00000" />
    <atom id="a2" elementType="C" x3="1.085779" y3="-2.318681" z3="0.00000" />
    <atom id="a3" elementType="H" x3="-1.188069" y3="-2.318681" z3="0.00000" />
    <atom id="a4" elementType="H" x3="2.147511" y3="-2.318681" z3="0.00000" />
  </atomArray>
  <bondArray>
    <bond atomRefs2="a1 a2" order="3" />
    <bond atomRefs2="a1 a3" order="1" />
    <bond atomRefs2="a2 a4" order="1" />
  </bondArray>
  <propertyList>
    <property dictRef="me:ZPE">
      <scalar units="kJ/mol">0</scalar>
    </property>
    <property dictRef="me:frequenciesScaleFactor">
      <scalar>0.953</scalar>
    </property>
    <property dictRef="me:vibFreqs">
      <array units="cm-1">601.3199 601.3199 753.8609 753.8609 1967.8951 3431.6999
      3533.7308</array>
    </property>
    <property dictRef="me:rotConsts">
      <array units="cm-1">1.168840912 1.168840912</array>
    </property>
    <property dictRef="me:symmetryNumber">
      <scalar>1</scalar>
    </property>
    <property dictRef="me:MW">
      <scalar units="amu">26.04</scalar>
    </property>
    <property dictRef="me:spinMultiplicity">
      <scalar>1</scalar>
    </property>
    <property dictRef="me:epsilon">
      <scalar>300</scalar>
    </property>
    <property dictRef="me:sigma">
      <scalar>4</scalar>
    </property>
  </propertyList>
  <me:DOSCMethod xsi:type="QMRotors" />
  <me:energyTransferModel xsi:type="me:ExponentialDown">
    <me:deltaEDown bathGas="N2" units="cm-1">250</me:deltaEDown>
    <me:deltaEDownTExponent bathGas="N2" referenceTemperature="298">0.25
    </me:deltaEDownTExponent>
    <me:deltaEDown bathGas="Ar" units="cm-1">200</me:deltaEDown>
    <me:deltaEDownTExponent bathGas="Ar" referenceTemperature="298">0.5
    </me:deltaEDownTExponent>
    <me:deltaEDown bathGas="He" units="cm-1">100</me:deltaEDown>
    <me:deltaEDownTExponent bathGas="He" referenceTemperature="298">1.0
    </me:deltaEDownTExponent>
  </me:energyTransferModel>
</molecule>

<molecule id="CO">
  <atomArray>
    <atom id="a1" elementType="C" x3="2.091427" y3="-0.880314" z3="-0.425302" />
    <atom id="a2" elementType="O" x3="1.659995" y3="0.033987" z3="-0.949846" />
  </atomArray>
  <bondArray>
    <bond atomRefs2="a1 a2" order="3" />
  </bondArray>
  <propertyList>
    <property dictRef="me:ZPE">
      <!-- <scalar units="kJ/mol">-143.5043 </scalar> -->
      <scalar units="kJ/mol">-143.5</scalar>
    </property>
    <property dictRef="me:frequenciesScaleFactor">
      <scalar>0.953</scalar>
    </property>
```

```
          <property dictRef="me:vibFreqs">
            <array units="cm-1">2109.678</array>
          </property>
          <property dictRef="me:rotConsts">
            <array units="cm-1">1.895381771 1.895381771</array>
          </property>
          <property dictRef="me:symmetryNumber">
            <scalar>1</scalar>
          </property>
          <property dictRef="me:MW">
            <scalar units="amu">28</scalar>
          </property>
          <property dictRef="me:spinMultiplicity">
            <scalar>1</scalar>
          </property>
          <property dictRef="me:epsilon">
            <scalar>300</scalar>
          </property>
          <property dictRef="me:sigma">
            <scalar>4</scalar>
          </property>
        </propertyList>
        <me:DOSCMethod xsi:type="QMRotors" />
        <me:energyTransferModel xsi:type="me:ExponentialDown">
          <me:deltaEDown bathGas="N2" units="cm-1">250</me:deltaEDown>
          <me:deltaEDownTExponent bathGas="N2" referenceTemperature="298">0.25
          </me:deltaEDownTExponent>
          <me:deltaEDown bathGas="Ar" units="cm-1">200</me:deltaEDown>
          <me:deltaEDownTExponent bathGas="Ar" referenceTemperature="298">0.5
          </me:deltaEDownTExponent>
          <me:deltaEDown bathGas="He" units="cm-1">100</me:deltaEDown>
          <me:deltaEDownTExponent bathGas="He" referenceTemperature="298">1.0
          </me:deltaEDownTExponent>
        </me:energyTransferModel>
      </molecule>

      <molecule id="H">
        <atom elementType="H" />
        <propertyList>
          <property dictRef="me:ZPE">
            <scalar units="cm-1">0.0</scalar>
          </property>
          <property dictRef="me:MW">
            <scalar>1.01</scalar>
          </property>
          <property dictRef="me:spinMultiplicity">
            <scalar>2</scalar>
          </property>
          <property dictRef="me:epsilon">
            <scalar>50</scalar>
          </property>
          <property dictRef="me:sigma">
            <scalar>2</scalar>
          </property>
        </propertyList>
      </molecule>

      <molecule id="CHO-TS">
        <atomArray>
          <atom id="a1" elementType="C" x3="1.859219" y3="-0.794856" z3="-0.367817" />
          <atom id="a2" elementType="H" x3="3.434012" y3="-1.499480" z3="-0.653161" />
          <atom id="a3" elementType="O" x3="1.569739" y3="0.127596" z3="-0.977968" />
        </atomArray>
        <bondArray>
          <bond atomRefs2="a1 a2" order="1" />
          <bond atomRefs2="a1 a3" order="2" />
        </bondArray>
        <propertyList>
          <property dictRef="me:ZPE">
            <scalar units="kJ/mol">-130.0 </scalar>
            <!-- <scalar units="kJ/mol" lower="-7.5" upper="2.5"
            stepsize="0.05">-2.5521 </scalar> -->
```

```
590           </property>
591           <property dictRef="me:frequenciesScaleFactor">
592             <scalar>0.953</scalar>
593           </property>
594           <property dictRef="me:vibFreqs">
595             <array units="cm-1">9.359777823 1.455057285 1.259289852</array>
596           </property>
597           <property dictRef="me:rotConsts">
598             <array units="cm-1">472.2134 2080.7418</array>
599           </property>
600           <property title="ImaginaryFrequency" dictRef="me:imFreqs">
601             <scalar units="cm-1">1071.8735</scalar>
602             <!-- <scalar units="cm-1" lower="100" upper="1000" stepsize="5">-219.4242
              </scalar> -->
603           </property>
604           <property dictRef="me:symmetryNumber">
605             <scalar>1</scalar>
606           </property>
607           <property dictRef="me:MW">
608             <scalar units="amu">29.02</scalar>
609           </property>
610           <property dictRef="me:spinMultiplicity">
611             <scalar>2</scalar>
612           </property>
613           <property dictRef="me:epsilon">
614             <scalar>250</scalar>
615           </property>
616           <property dictRef="me:sigma">
617             <scalar>4.2</scalar>
618           </property>
619         </propertyList>
620         <me:DOSCMethod xsi:type="QMRotors" />
621         <me:energyTransferModel xsi:type="me:ExponentialDown">
622           <me:deltaEDown bathGas="N2" units="cm-1">250</me:deltaEDown>
623           <me:deltaEDownTExponent bathGas="N2" referenceTemperature="298">0.25
              </me:deltaEDownTExponent>
624           <me:deltaEDown bathGas="Ar" units="cm-1">200</me:deltaEDown>
625           <me:deltaEDownTExponent bathGas="Ar" referenceTemperature="298">0.5
              </me:deltaEDownTExponent>
626           <me:deltaEDown bathGas="He" units="cm-1">100</me:deltaEDown>
627           <me:deltaEDownTExponent bathGas="He" referenceTemperature="298">1.0
              </me:deltaEDownTExponent>
628         </me:energyTransferModel>
629         <me:densityOfStatesList calculated="20231201_135454">
630           <me:description>Rovibronic partition function calculation at various
              temperatures. qtot : product of QM partition functions for vibrations (1-D
              harmonic oscillator) and classical partition functions for rotations.  sumc :
              cell based partition function. sumg : grain based partition function
              </me:description>
631           <me:densityOfStates>
632             <me:T>200</me:T>
633             <me:qtot>25194.6</me:qtot>
634             <me:sumc>618646</me:sumc>
635             <me:sumg>617453</me:sumg>
636           </me:densityOfStates>
637           <me:densityOfStates>
638             <me:T>300</me:T>
639             <me:qtot>125812</me:qtot>
640             <me:sumc>2.06325e+06</me:sumc>
641             <me:sumg>2.06148e+06</me:sumg>
642           </me:densityOfStates>
643           <me:densityOfStates>
644             <me:T>400</me:T>
645             <me:qtot>394910</me:qtot>
646             <me:sumc>4.86161e+06</me:sumc>
647             <me:sumg>4.85926e+06</me:sumg>
648           </me:densityOfStates>
649         </me:densityOfStatesList>
650       </molecule>
651
652       <molecule id="C-add-TS">
653         <atomArray>
```

```
654           <atom id="a1" elementType="C" x3="-1.435998" y3="0.585996" z3="0.055906" />
655           <atom id="a2" elementType="H" x3="-0.975420" y3="-0.386516" z3="-0.184991" />
656           <atom id="a3" elementType="H" x3="-0.786107" y3="1.466369" z3="-0.080207" />
657           <atom id="a4" elementType="O" x3="-2.530748" y3="0.670634" z3="0.537137" />
658           <atom id="a5" elementType="C" x3="-2.131019" y3="0.774404" z3="-2.020007" />
659           <atom id="a6" elementType="H" x3="-2.298467" y3="0.857099" z3="-3.179730" />
660           <atom id="a7" elementType="C" x3="-2.436892" y3="0.930586" z3="-4.229081" />
661         </atomArray>
662         <bondArray>
663           <bond atomRefs2="a1 a2" order="1" />
664           <bond atomRefs2="a1 a3" order="1" />
665           <bond atomRefs2="a1 a4" order="2" />
666           <bond atomRefs2="a1 a5" order="1" />
667           <bond atomRefs2="a5 a6" order="3" />
668           <bond atomRefs2="a7 a6" order="1" />
669         </bondArray>
670         <propertyList>
671           <property dictRef="me:ZPE">
672             <scalar units="kJ/mol">118.9470</scalar>
673             <!-- <scalar units="kJ/mol" lower="-7.5" upper="2.5" stepsize="0.05">-5.35
              </scalar> -->
674             <!-- (108.42 M0622X, 15.06 MP2_Dong, or 12.13 QCISD_Dong) -->
675           </property>
676           <property dictRef="me:frequenciesScaleFactor">
677             <scalar>0.953</scalar>
678           </property>
679           <property dictRef="me:vibFreqs">
680             <array units="cm-1">93.8891 106.0246 225.1745 446.8415 852.3411 860.3819
              1201.4456 1271.7515 1547.0691 1775.6663 2562.9914 2952.0146 3025.8317
              3566.0523</array>
681           </property>
682           <property dictRef="me:rotConsts">
683             <array units="cm-1">1.2627 0.1247 0.1162</array>
684           </property>
685           <property title="ImaginaryFrequency" dictRef="me:imFreqs">
686             <scalar units="cm-1">497.0607</scalar>
687             <!-- <scalar units="cm-1" lower="50" upper="2000" stepsize="5">219.4242
              </scalar> -->
688           </property>
689           <property dictRef="me:symmetryNumber">
690             <scalar>1</scalar>
691           </property>
692           <property dictRef="me:MW">
693             <scalar units="amu">55.02</scalar>
694           </property>
695           <property dictRef="me:spinMultiplicity">
696             <scalar>2</scalar>
697           </property>
698           <property dictRef="me:epsilon">
699             <scalar>350</scalar>
700           </property>
701           <property dictRef="me:sigma">
702             <scalar>5</scalar>
703           </property>
704         </propertyList>
705         <me:DOSCMethod xsi:type="QMRotors" />
706         <me:energyTransferModel xsi:type="me:ExponentialDown">
707           <me:deltaEDown bathGas="N2" units="cm-1">250</me:deltaEDown>
708           <me:deltaEDownTExponent bathGas="N2" referenceTemperature="298">0.25
              </me:deltaEDownTExponent>
709           <me:deltaEDown bathGas="Ar" units="cm-1">200</me:deltaEDown>
710           <me:deltaEDownTExponent bathGas="Ar" referenceTemperature="298">0.5
              </me:deltaEDownTExponent>
711           <me:deltaEDown bathGas="He" units="cm-1">100</me:deltaEDown>
712           <me:deltaEDownTExponent bathGas="He" referenceTemperature="298">1.0
              </me:deltaEDownTExponent>
713         </me:energyTransferModel>
714       </molecule>
715
716       <molecule id="C-add">
717         <atomArray>
718           <atom id="a1" elementType="C" x3="-1.413884" y3="0.610162" z3="-0.409292" />
```

```
719           <atom id="a2" elementType="H" x3="-0.920766" y3="-0.332784" z3="-0.141976" />
720           <atom id="a3" elementType="H" x3="-0.740683" y3="1.398514" z3="-0.050030" />
721           <atom id="a4" elementType="O" x3="-2.570007" y3="0.690857" z3="0.335351" />
722           <atom id="a5" elementType="C" x3="-1.588380" y3="0.705522" z3="-1.863122" />
723           <atom id="a6" elementType="C" x3="-1.716557" y3="0.782708" z3="-3.065433" />
724           <atom id="a7" elementType="H" x3="-1.839681" y3="0.851411" z3="-4.117919" />
725         </atomArray>
726         <bondArray>
727           <bond atomRefs2="a1 a2" order="1" />
728           <bond atomRefs2="a1 a3" order="1" />
729           <bond atomRefs2="a1 a4" order="2" />
730           <bond atomRefs2="a1 a5" order="1" />
731           <bond atomRefs2="a5 a6" order="3" />
732           <bond atomRefs2="a6 a7" order="1" />
733         </bondArray>
734         <propertyList>
735           <property dictRef="me:ZPE">
736             <scalar units="kJ/mol">-186.8904</scalar>
737             <!-- <scalar units="kJ/mol" lower="-10.0" upper="-0.5" stepsize="0.10">-255
              </scalar> -->
738           </property>
739           <property dictRef="me:frequenciesScaleFactor">
740             <scalar>0.953</scalar>
741           </property>
742           <property dictRef="me:vibFreqs">
743             <array units="cm-1">205.2431 288.4995 500.9172 571.0997 667.7524 692.1933
              936.8686 1069.473 1163.0728 1357.1724 1416.4552 2347.4208 3028.8185
              3061.714 3501.902</array>
744           </property>
745           <property dictRef="me:rotConsts">
746             <array units="cm-1">1.354041068 0.156543965 0.143991281</array>
747           </property>
748           <property dictRef="me:symmetryNumber">
749             <scalar>1</scalar>
750           </property>
751           <property dictRef="me:MW">
752             <scalar units="amu">55.02</scalar>
753           </property>
754           <property dictRef="me:spinMultiplicity">
755             <scalar>2</scalar>
756           </property>
757           <property dictRef="me:epsilon">
758             <scalar>350</scalar>
759           </property>
760           <property dictRef="me:sigma">
761             <scalar>5</scalar>
762           </property>
763         </propertyList>
764         <me:DOSCMethod xsi:type="QMRotors" />
765         <me:energyTransferModel xsi:type="me:ExponentialDown">
766           <me:deltaEDown bathGas="N2" units="cm-1">150</me:deltaEDown>
767           <me:deltaEDownTExponent bathGas="N2" referenceTemperature="298">0.0
              </me:deltaEDownTExponent>
768           <me:deltaEDown bathGas="Ar" units="cm-1">100</me:deltaEDown>
769           <me:deltaEDownTExponent bathGas="Ar" referenceTemperature="298">0.0
              </me:deltaEDownTExponent>
770           <me:deltaEDown bathGas="He" units="cm-1">50</me:deltaEDown>
771           <me:deltaEDownTExponent bathGas="He" referenceTemperature="298">0.0
              </me:deltaEDownTExponent>
772         </me:energyTransferModel>
773       </molecule>
774
775       <molecule id="O-add-TS">
776         <atomArray>
777           <atom id="a1" elementType="C" x3="-2.324857" y3="0.470463" z3="0.099839" />
778           <atom id="a2" elementType="H" x3="-2.012442" y3="-0.574187" z3="0.006903" />
779           <atom id="a3" elementType="H" x3="-2.005107" y3="1.064691" z3="0.965067" />
780           <atom id="a4" elementType="O" x3="-3.024154" y3="0.969367" z3="-0.754093" />
781           <atom id="a5" elementType="C" x3="-3.409283" y3="2.804737" z3="-0.323391" />
782           <atom id="a6" elementType="H" x3="-3.004398" y3="3.357599" z3="0.656984" />
783           <atom id="a7" elementType="C" x3="-2.749183" y3="4.012734" z3="1.451990" />
784         </atomArray>
```

```
      <bondArray>
        <bond atomRefs2="a1 a2" order="1" />
        <bond atomRefs2="a1 a3" order="1" />
        <bond atomRefs2="a1 a4" order="2" />
        <bond atomRefs2="a4 a5" order="1" />
        <bond atomRefs2="a5 a6" order="3" />
        <bond atomRefs2="a7 a6" order="1" />
      </bondArray>
      <propertyList>
        <property dictRef="me:ZPE">
          <scalar units="kJ/mol">12.7867</scalar>
          <!-- <scalar units="kJ/mol" lower="-7.5" upper="2.5" stepsize="0.05">-5.35
          </scalar> -->
          <!-- (108.42 M062X, 15.06 MP2_Dong, 12.13 QCISD_Dong) -->
        </property>
        <property dictRef="me:frequenciesScaleFactor">
          <scalar>0.953</scalar>
        </property>
        <property dictRef="me:vibFreqs">
          <array units="cm-1">74.6103 267.4348 279.6804 475.7587 750.529 888.3267
          1250.6195 1263.7738 1494.0546 2169.4025 2473.1554 3055.077 3129.1877
          3539.9752</array>
        </property>
        <property dictRef="me:rotConsts">
          <array units="cm-1">0.887302842 0.181133976 0.150426066</array>
        </property>
        <property title="ImaginaryFrequency" dictRef="me:imFreqs">
          <scalar units="cm-1">239.3839</scalar>
          <!-- <scalar units="cm-1" lower="50" upper="2000" stepsize="5">219.4242
          </scalar> -->
        </property>
        <property dictRef="me:symmetryNumber">
          <scalar>1</scalar>
        </property>
        <property dictRef="me:MW">
          <scalar units="amu">55.02</scalar>
        </property>
        <property dictRef="me:spinMultiplicity">
          <scalar>2</scalar>
        </property>
        <property dictRef="me:epsilon">
          <scalar>350</scalar>
        </property>
        <property dictRef="me:sigma">
          <scalar>5</scalar>
        </property>
      </propertyList>
      <me:DOSCMethod xsi:type="QMRotors" />
      <me:energyTransferModel xsi:type="me:ExponentialDown">
        <me:deltaEDown bathGas="N2" units="cm-1">250</me:deltaEDown>
        <me:deltaEDownTExponent bathGas="N2" referenceTemperature="298">0.25
        </me:deltaEDownTExponent>
        <me:deltaEDown bathGas="Ar" units="cm-1">200</me:deltaEDown>
        <me:deltaEDownTExponent bathGas="Ar" referenceTemperature="298">0.5
        </me:deltaEDownTExponent>
        <me:deltaEDown bathGas="He" units="cm-1">100</me:deltaEDown>
        <me:deltaEDownTExponent bathGas="He" referenceTemperature="298">1.0
        </me:deltaEDownTExponent>
      </me:energyTransferModel>
    </molecule>

    <molecule id="O-add">
      <atomArray>
        <atom id="a1" elementType="C" x3="-1.946188" y3="0.478203" z3="-0.041256" />
        <atom id="a2" elementType="H" x3="-1.954654" y3="-0.586362" z3="-0.184646" />
        <atom id="a3" elementType="H" x3="-1.176401" y3="1.009432" z3="0.493513" />
        <atom id="a4" elementType="O" x3="-3.221701" y3="1.008178" z3="0.009027" />
        <atom id="a5" elementType="C" x3="-3.295274" y3="2.274699" z3="0.332459" />
        <atom id="a6" elementType="C" x3="-3.403727" y3="3.442578" z3="0.632815" />
        <atom id="a7" elementType="H" x3="-3.508456" y3="4.465102" z3="0.891144" />
      </atomArray>
      <bondArray>
```

```
850           <bond atomRefs2="a1 a2" order="1" />
851           <bond atomRefs2="a1 a3" order="1" />
852           <bond atomRefs2="a1 a4" order="2" />
853           <bond atomRefs2="a4 a5" order="1" />
854           <bond atomRefs2="a5 a6" order="3" />
855           <bond atomRefs2="a6 a7" order="1" />
856         </bondArray>
857         <propertyList>
858           <property dictRef="me:ZPE">
859             <scalar units="kJ/mol">-156.0515</scalar>
860             <!-- <scalar units="kJ/mol" lower="-10.0" upper="-0.5" stepsize="0.10">-255
              </scalar> -->
861           </property>
862           <property dictRef="me:frequenciesScaleFactor">
863             <scalar>0.953</scalar>
864           </property>
865           <property dictRef="me:vibFreqs">
866             <array units="cm-1">213.9642 241.8212 385.4406 540.7205 547.5639 591.5609
              618.1073 954.8054 1195.7795 1260.3723 1479.133 2211.0553 3205.3643
              3372.0472 3504.3344</array>
867           </property>
868           <property dictRef="me:rotConsts">
869             <array units="cm-1">1.628968264 0.177108525 0.160026708</array>
870           </property>
871           <property dictRef="me:symmetryNumber">
872             <scalar>1</scalar>
873           </property>
874           <property dictRef="me:MW">
875             <scalar units="amu">55.02</scalar>
876           </property>
877           <property dictRef="me:spinMultiplicity">
878             <scalar>2</scalar>
879           </property>
880           <property dictRef="me:epsilon">
881             <scalar>350</scalar>
882           </property>
883           <property dictRef="me:sigma">
884             <scalar>5</scalar>
885           </property>
886         </propertyList>
887         <me:DOSCMethod xsi:type="QMRotors" />
888         <me:energyTransferModel xsi:type="me:ExponentialDown">
889           <me:deltaEDown bathGas="N2" units="cm-1">150</me:deltaEDown>
890           <me:deltaEDownTExponent bathGas="N2" referenceTemperature="298">0.0
              </me:deltaEDownTExponent>
891           <me:deltaEDown bathGas="Ar" units="cm-1">100</me:deltaEDown>
892           <me:deltaEDownTExponent bathGas="Ar" referenceTemperature="298">0.0
              </me:deltaEDownTExponent>
893           <me:deltaEDown bathGas="He" units="cm-1">50</me:deltaEDown>
894           <me:deltaEDownTExponent bathGas="He" referenceTemperature="298">0.0
              </me:deltaEDownTExponent>
895         </me:energyTransferModel>
896       </molecule>
897
898       <molecule id="N2">
899         <propertyList>
900           <property dictRef="me:epsilon">
901             <scalar>82.0</scalar>
902           </property>
903           <property dictRef="me:sigma">
904             <scalar>3.74</scalar>
905           </property>
906           <property dictRef="me:MW">
907             <scalar units="amu">28.0</scalar>
908           </property>
909         </propertyList>
910       </molecule>
911
912       <molecule id="He">
913         <propertyList>
914           <property dictRef="me:epsilon">
915             <scalar>10.22</scalar>
```

```
916           </property>
917           <property dictRef="me:sigma">
918             <scalar>2.511</scalar>
919           </property>
920           <property dictRef="me:MW">
921             <scalar>4.04</scalar>
922           </property>
923         </propertyList>
924       </molecule>
925
926       <molecule id="Ar">
927         <propertyList>
928           <property dictRef="me:epsilon">
929             <scalar>114</scalar>
930           </property>
931           <property dictRef="me:sigma">
932             <scalar>3.47</scalar>
933           </property>
934           <property dictRef="me:MW">
935             <scalar>39.948</scalar>
936           </property>
937         </propertyList>
938       </molecule>
939
940       <molecule id="O2">
941         <propertyList>
942           <property dictRef="me:epsilon">
943             <scalar>103</scalar>
944           </property>
945           <property dictRef="me:sigma">
946             <scalar>3.48</scalar>
947           </property>
948           <property dictRef="me:MW">
949             <scalar units="amu">32.0</scalar>
950           </property>
951         </propertyList>
952       </molecule>
953
954       <molecule id="SF6">
955         <propertyList>
956           <property dictRef="me:epsilon">
957             <scalar>212</scalar>
958           </property>
959           <property dictRef="me:sigma">
960             <scalar>5.20</scalar>
961           </property>
962           <property dictRef="me:MW">
963             <scalar>146</scalar>
964           </property>
965         </propertyList>
966       </molecule>
967
968     </moleculeList>
969
970     <reactionList>
971
972       <reaction id="R1" reversible="true">
973         <reactant>
974           <molecule ref="C2H" role="modelled" />
975         </reactant>
976         <reactant>
977           <molecule ref="CH2O" role="excessReactant" />
978         </reactant>
979         <product>
980           <molecule ref="PRC" role="modelled" />
981         </product>
982         <me:MCRCMethod xsi:type="me:MesmerILT">
983           <me:preExponential units="cm3molecule-1s-1">1.20e-10 </me:preExponential>
984           <!-- <me:preExponential units="cm3molecule-1s-1" lower="5.0e-12"
              upper="8.0e-10" stepsize="2e-12">1.2e-10</me:preExponential> -->
985           <me:activationEnergy units="kJ/mol">0.0</me:activationEnergy>
986           <!-- <me:nInfinity lower="-1.4" upper="1.4" stepsize="0.005">0.0
```

```
            </me:nInfinity> -->
            <me:TInfinity>298.0</me:TInfinity>
            <me:nInfinity>-0.04 </me:nInfinity>
            <!-- <me:nInfinity lower="-1.5" upper="1.5"
            stepsize="0.02">-0.04</me:nInfinity> -->
          </me:MCRCMethod>
          <me:excessReactantConc>1E13</me:excessReactantConc>
          <!-- (put in a high excess to get branching ratio correct - say 1e20 - will
          give wrong k if P is lower than excess though) -->
          <me:FragmentDist name="Prior" default="true" />
        </reaction>

        <!-- <reaction id="R2"> -->
        <!-- <reactant> -->
        <!-- <molecule ref="PRC" role="modelled" /> -->
        <!-- </reactant> -->
        <!-- <product> -->
        <!-- <molecule ref="CHO" role="sink" /> -->
        <!-- </product> -->
        <!-- <product> -->
        <!-- <molecule ref="C2H2" role="sink" /> -->
        <!-- </product> -->
        <!-- <me:transitionState> -->
        <!-- <molecule ref="TS" role="transitionState" /> -->
        <!-- </me:transitionState> -->
        <!-- <me:tunneling>Eckart </me:tunneling>  -->
        <!-- <me:MCRCMethod name="RRKM"/> -->
        <!-- </reaction> -->

        <reaction id="R2">
          <reactant>
            <molecule ref="PRC" role="modelled" />
          </reactant>
          <product>
            <molecule ref="PostRC" role="modelled" />
          </product>
          <me:transitionState>
            <molecule ref="TS" role="transitionState" />
          </me:transitionState>
          <me:tunneling>Eckart</me:tunneling>
          <me:MCRCMethod name="RRKM" />
        </reaction>

        <reaction id="R3" reverse="true">
          <product>
            <molecule ref="PostRC" me:type="modelled" />
          </product>
          <reactant>
            <molecule ref="CHO" me:type="modelled" />
          </reactant>
          <reactant>
            <molecule ref="C2H2" me:type="excessReactant" />
          </reactant>
          <me:MCRCMethod xsi:type="me:MesmerILT">
            <me:preExponential units="cm3molecule-1s-1">1e-10</me:preExponential>
            <me:activationEnergy units="kJ/mol">0.0</me:activationEnergy>
            <me:TInfinity>298.0</me:TInfinity>
            <me:nInfinity>0</me:nInfinity>
          </me:MCRCMethod>
          <me:excessReactantConc>1E20</me:excessReactantConc>
          <me:FragmentDist xsi:type="me:Prior">
            <me:WriteDistribution />
          </me:FragmentDist>
        </reaction>

        <reaction id="R4">
          <reactant>
            <molecule ref="CHO" role="modelled" />
          </reactant>
          <product>
            <molecule ref="H" role="sink" />
          </product>
```

```
1056        <product>
1057          <molecule ref="CO" role="sink" />
1058        </product>
1059        <me:transitionState>
1060          <molecule ref="CHO-TS" role="transitionState" />
1061        </me:transitionState>
1062        <me:tunneling>Eckart</me:tunneling>
1063        <me:MCRCMethod name="RRKM" />
1064      </reaction>
1065
1066      <!-- <reaction id="R5"> -->
1067        <!-- <reactant> -->
1068          <!-- <molecule ref="PRC" role="modelled" /> -->
1069        <!-- </reactant> -->
1070        <!-- <product> -->
1071          <!-- <molecule ref="C-add" role="sink" /> -->
1072        <!-- </product> -->
1073        <!-- <me:transitionState> -->
1074          <!-- <molecule ref="C-add-TS" role="transitionState" /> -->
1075        <!-- </me:transitionState> -->
1076        <!-- <me:tunneling>Eckart </me:tunneling>  -->
1077        <!-- <me:MCRCMethod name="RRKM"/> -->
1078      <!-- </reaction> -->
1079
1080      <!-- <reaction id="R6"> -->
1081        <!-- <reactant> -->
1082          <!-- <molecule ref="PRC" role="modelled" /> -->
1083        <!-- </reactant> -->
1084        <!-- <product> -->
1085          <!-- <molecule ref="O-add" role="sink" /> -->
1086        <!-- </product> -->
1087        <!-- <me:transitionState> -->
1088          <!-- <molecule ref="O-add-TS" role="transitionState" /> -->
1089        <!-- </me:transitionState> -->
1090        <!-- <me:tunneling>Eckart </me:tunneling>  -->
1091        <!-- <me:MCRCMethod name="RRKM"/> -->
1092      <!-- </reaction>     -->
1093
1094    </reactionList>
1095
1096    <me:conditions>
1097
1098      <me:InitialPopulation>
1099        <me:molecule ref="C2H" me:population="1.0" />
1100      </me:InitialPopulation>
1101
1102      <me:bathGas>N2</me:bathGas>
1103
1104      <me:PTs>
1105
1106        <!-- Put in the psuedo first order rate constant -->
1107
1108        <!-- <me:PTpair units="PPCC" P="5.09e16" T="37.2" me:precision="dd">  -->
1109        <!-- <me:bathGas>He </me:bathGas>  -->
1110        <!-- <me:experimentalRate ref1="C2H" ref2="C2H"
          error="80">1170</me:experimentalRate> -->
1111        <!-- </me:PTpair> -->
1112
1113        <!-- <me:PTpair units="PPCC" P="5.09e16" T="37.2" me:precision="dd">  -->
1114        <!-- <me:bathGas>He </me:bathGas>  -->
1115        <!-- <me:experimentalRate ref1="C2H" ref2="C2H"
          error="200">1390</me:experimentalRate> -->
1116        <!-- </me:PTpair> -->
1117
1118        <!-- <me:PTpair units="PPCC" P="9.32e16" T="43.7" me:precision="dd">  -->
1119        <!-- <me:bathGas>He </me:bathGas>  -->
1120        <!-- <me:experimentalRate ref1="C2H" ref2="C2H"
          error="380">2030</me:experimentalRate> -->
1121        <!-- </me:PTpair> -->
1122
1123        <!-- <me:PTpair units="PPCC" P="7.12e16" T="50.5" me:precision="dd">  -->
1124        <!-- <me:bathGas>He </me:bathGas>  -->
```

```
1125        <!-- <me:experimentalRate ref1="C2H" ref2="C2H"
            error="60">890</me:experimentalRate> -->
1126        <!-- </me:PTpair> -->
1127
1128        <!-- <me:PTpair units="PPCC" P="9.26e16" T="60.7" me:precision="dd">  -->
1129        <!-- <me:bathGas>He </me:bathGas>  -->
1130        <!-- <me:experimentalRate ref1="C2H" ref2="C2H"
            error="100">1440</me:experimentalRate> -->
1131        <!-- </me:PTpair> -->
1132
1133        <!-- <me:PTpair units="PPCC" P="2.85e16" T="67.0" me:precision="dd">  -->
1134        <!-- <me:bathGas>N2 </me:bathGas>  -->
1135        <!-- <me:experimentalRate ref1="C2H" ref2="C2H"
            error="90">911</me:experimentalRate> -->
1136        <!-- </me:PTpair>  -->
1137
1138        <!-- <me:PTpair units="PPCC" P="7.44e16" T="83.4" me:precision="dd">  -->
1139        <!-- <me:bathGas>N2 </me:bathGas>  -->
1140        <!-- <me:experimentalRate ref1="C2H" ref2="C2H"
            error="90">721</me:experimentalRate> -->
1141        <!-- </me:PTpair>  -->
1142
1143        <!-- <me:PTpair units="PPCC" P="7.44e16" T="83.4" me:precision="dd">  -->
1144        <!-- <me:bathGas>N2 </me:bathGas>  -->
1145        <!-- <me:experimentalRate ref1="C2H" ref2="C2H"
            error="70">1020</me:experimentalRate> -->
1146        <!-- </me:PTpair> -->
1147
1148        <!-- <me:PTpair units="PPCC" P="5.33e16" T="92.8" me:precision="dd">  -->
1149        <!-- <me:bathGas>N2 </me:bathGas>  -->
1150        <!-- <me:experimentalRate ref1="C2H" ref2="C2H"
            error="100">727</me:experimentalRate> -->
1151        <!-- </me:PTpair> -->
1152
1153        <!-- <me:PTpair units="PPCC" P="5.33e16" T="92.8" me:precision="dd">  -->
1154        <!-- <me:bathGas>N2 </me:bathGas>  -->
1155        <!-- <me:experimentalRate ref1="C2H" ref2="C2H"
            error="100">793</me:experimentalRate> -->
1156        <!-- </me:PTpair> -->
1157
1158        <!-- <me:PTpair units="PPCC" P="5.33e16" T="308" me:precision="dd">  -->
1159        <!-- <me:bathGas>Ar </me:bathGas>  -->
1160        <!-- <me:experimentalRate ref1="C2H" ref2="C2H"
            error="47">470</me:experimentalRate> -->
1161        <!-- </me:PTpair> -->
1162
1163        <!-- <me:PTpair units="PPCC" P="8.30e17" T="603" me:precision="dd">  -->
1164        <!-- <me:bathGas>Ar </me:bathGas>  -->
1165        <!-- <me:experimentalRate ref1="C2H" ref2="C2H"
            error="60.3">603</me:experimentalRate> -->
1166        <!-- </me:PTpair> -->
1167
1168        <!-- <me:PTpair units="PPCC" P="8.30e17" T="603" me:precision="dd">  -->
1169        <!-- <me:bathGas>Ar </me:bathGas>  -->
1170        <!-- <me:experimentalRate ref1="C2H" ref2="C2H"
            error="55.8">558</me:experimentalRate> -->
1171        <!-- </me:PTpair> -->
1172
1173        <me:PTpair me:units="PPCC" me:P="1e19" me:T="600" me:precision="dd" bathGas="N2"
             group="default" />
1174        <me:PTpair me:units="PPCC" me:P="1e19" me:T="500" me:precision="dd" bathGas="N2"
             group="default" />
1175        <me:PTpair me:units="PPCC" me:P="1e19" me:T="400" me:precision="dd" bathGas="N2"
             group="default" />
1176        <me:PTpair me:units="PPCC" me:P="1e19" me:T="300" me:precision="dd" bathGas="N2"
             group="default" />
1177        <!-- <me:PTpair me:units="PPCC" me:P="1e19" me:T="275" me:precision="dd"
            bathGas="N2" group="default" /> -->
1178        <!-- <me:PTpair me:units="PPCC" me:P="1e19" me:T="250" me:precision="dd"
            bathGas="N2" group="default" /> -->
1179        <!-- <me:PTpair me:units="PPCC" me:P="1e19" me:T="225" me:precision="dd"
            bathGas="N2" group="default" /> -->
```

```
1180        <!-- <me:PTpair me:units="PPCC" me:P="1e19" me:T="200" me:precision="dd"
            bathGas="N2" group="default" /> -->
1181        <!-- <me:PTpair me:units="PPCC" me:P="1e19" me:T="175" me:precision="qd"
            bathGas="N2" group="default" /> -->
1182        <!-- <me:PTpair me:units="PPCC" me:P="1e19" me:T="150" me:precision="qd"
            bathGas="N2" group="default" /> -->
1183
1184        <me:PTpair me:units="PPCC" me:P="1e11" me:T="600" me:precision="dd" bathGas="N2"
             group="default" />
1185        <me:PTpair me:units="PPCC" me:P="1e11" me:T="500" me:precision="dd" bathGas="N2"
             group="default" />
1186        <me:PTpair me:units="PPCC" me:P="1e11" me:T="400" me:precision="dd" bathGas="N2"
             group="default" />
1187        <me:PTpair me:units="PPCC" me:P="1e11" me:T="300" me:precision="dd" bathGas="N2"
             group="default" />
1188        <!-- <me:PTpair me:units="PPCC" me:P="1e11" me:T="275" me:precision="dd"
            bathGas="N2" group="default" /> -->
1189        <!-- <me:PTpair me:units="PPCC" me:P="1e11" me:T="250" me:precision="dd"
            bathGas="N2" group="default" /> -->
1190        <!-- <me:PTpair me:units="PPCC" me:P="1e11" me:T="225" me:precision="dd"
            bathGas="N2" group="default" /> -->
1191        <!-- <me:PTpair me:units="PPCC" me:P="1e11" me:T="200" me:precision="dd"
            bathGas="N2" group="default" /> -->
1192        <!-- <me:PTpair me:units="PPCC" me:P="1e11" me:T="175" me:precision="qd"
            bathGas="N2" group="default" /> -->
1193        <!-- <me:PTpair me:units="PPCC" me:P="1e11" me:T="150" me:precision="qd"
            bathGas="N2" group="default" /> -->
1194
1195      </me:PTs>
1196
1197    </me:conditions>
1198
1199    <me:modelParameters>
1200      <!-- Specify grain size directly...-->
1201      <me:grainSize units="cm-1">20</me:grainSize>
1202      <!-- ...or by the total number of grains...-->
1203      <!-- <me:numberOfGrains> 25 </me:numberOfGrains> -->
1204      <!-- Specify increased energy range -->
1205      <!-- <me:maxTemperature>6000</me:maxTemperature> -->
1206      <!-- <me:energyAboveTheTopHill>25.0 </me:energyAboveTheTopHill> -->
1207      <me:automaticallySetMaxEne>1e-15</me:automaticallySetMaxEne>
1208      <me:energyAboveTheTopHill units="kT" default="true">25</me:energyAboveTheTopHill>
1209    </me:modelParameters>
1210
1211    <me:control>
1212      <!-- <me:calcMethod xsi:type="me:marquardt"> -->
1213      <!-- <me:MarquardtIterations>10</me:MarquardtIterations> -->
1214      <!-- <me:MarquardtTolerance>1E-7</me:MarquardtTolerance> -->
1215      <!-- <me:MarquardtDerivDelta>0.025</me:MarquardtDerivDelta> -->
1216      <!-- </me:calcMethod> -->
1217      <!-- <me:calculateRateCoefficientsOnly /> -->
1218      <me:printSpeciesProfile />
1219      <me:eigenvalues>5</me:eigenvalues>
1220      <me:calcMethod default="true" name="simpleCalc" />
1221      <me:ForceMacroDetailedBalance default="true">true</me:ForceMacroDetailedBalance>
1222    </me:control>
1223
1224  </me:mesmer>
1225
```
